# Investigating multilevel cognitive processing within error-free and error-prone feedback conditions in executed and observed car driving

**DOI:** 10.3389/fnhum.2024.1383956

**Published:** 2024-06-27

**Authors:** Hannah S. Pulferer, Cuntai Guan, Gernot R. Müller-Putz

**Affiliations:** ^1^Institute of Neural Engineering, Graz University of Technology, Graz, Austria; ^2^College of Computing and Data Science, Nanyang Technological University, Singapore, Singapore; ^3^BioTechMed-Graz, Graz, Austria

**Keywords:** car driving, steering, electroencephalogram, error processing, feedback processing

## Abstract

Accident analyses repeatedly reported the considerable contribution of run-off-road incidents to fatalities in road traffic, and despite considerable advances in assistive technologies to mitigate devastating consequences, little insight into the drivers’ brain response during such accident scenarios has been gained. While various literature documents neural correlates to steering motion, the driver’s mental state, and the impact of distraction and fatigue on driving performance, the cortical substrate of continuous deviations of a car from the road – i.e., how the brain represents a varying discrepancy between the intended and observed car position and subsequently assigns customized levels of corrective measures – remains unclear. Furthermore, the superposition of multiple subprocesses, such as visual and erroneous feedback processing, performance monitoring, or motor control, complicates a clear interpretation of engaged brain regions within car driving tasks. In the present study, we thus attempted to disentangle these subprocesses, employing passive and active steering conditions within both error-free and error-prone vehicle operation conditions. We recorded EEG signals of 26 participants in 13 sessions, simultaneously measuring pairs of Executors (actively steering) and Observers (strictly observing) during a car driving task. We observed common brain patterns in the Executors regardless of error-free or error-prone vehicle operation, albeit with a shift in spectral activity from motor beta to occipital alpha oscillations within erroneous conditions. Further, significant frontocentral differences between Observers and Executors, tracing back to the caudal anterior cingulate cortex, arose during active steering conditions, indicating increased levels of motor-behavioral cognitive control. Finally, we present regression results of both the steering signal and the car position, indicating that a regression of continuous deviations from the road utilizing the EEG might be feasible.

## 1 Introduction

Driving a car constitutes a crucial part of everyday life for a large proportion of the population, and consequently, error situations like lane deviations are by no means rare occurrences in road traffic. Accident analyses unveiled that single-vehicle accidents comprising run-off-road, head-on, and sideswipe crashes contribute to a large portion of accident fatalities despite involving only a minor fraction of all occupants involved (Kusano and Gabler, 2014; Cicchino, 2018). However, while several engineering approaches, such as lane-keeping assistants, are well-researched and significantly contribute to decreasing accident and thus fatality rates (Kusano et al., 2014), little research has analyzed the drivers’ perception – i.e., the preceding and accompanying brain response – of erroneous vehicle behavior within these run-off-road scenarios.

Indeed, an abundance of non-invasive brain research investigated numerous aspects of vehicle operation to the present day. Various groups reported neural correlates to or successful classification of, e.g., steering motion (Simpson and Rafferty, 2022; Vecchiato et al., 2022), the driver’s mental state (ElSherif et al., 2020), or the influences of distraction and fatigue (Schneiders et al., 2020; Zeng et al., 2022) on the driving performance utilizing the electroencephalogram (EEG). Additionally, insights on discrete error-related brain activity occurring during the operation of a vehicle induced by wrong turns (Zhang et al., 2015) or steering errors caused by the interface (Garcia et al., 2017) emerged specifically in the context of brain-computer interfaces (BCIs).

While all of these and similar studies significantly contributed to in-depth documentation of cognitive responses relating to car driving, several issues and open questions remain unaddressed. For one, while the term car driving is often used to refer to the overall task of operating a vehicle, it can only be an umbrella term for the multitude of subprocesses transpiring simultaneously in a neuroscientific sense. Ranging from motor control and visual processing to performance monitoring, erroneous feedback processing, and possibly subsequent corrective behavior, brain recordings during car driving scenarios portray the full superposition and thus obscure the contribution of each subprocess to possible regression or classification successes. Furthermore, some of these subprocesses remain largely undocumented to the present day. While for example the occurrence of scalp potentials such as the frontocentral error-related negativity (ERN) (Falkenstein et al., 1989) and the centroparietal error positivity (Pe) (Nieuwenhuis et al., 2001) during vehicle operation is well-documented for various discrete error events (Wimmer et al., 2023), the neural representation of continuous deviations from the road – i.e., how the brain processes continuously varying discrepancies between intended and observed outcomes – remains largely unclear.

Previous source localization studies already tackled variations of these problems, utilizing functional magnetic resonance imaging and positron emission tomography to disentangle active steering and passive viewing of recorded car driving (Walter et al., 2001; Horikawa et al., 2005) and effectively separate the visual and motor-related components of driving. Garcia et al. (2017) identified distinct brain states related to proactive and reactive steering in an EEG study, differentiating generic and corrective motor output. Recent work further documented a neural correlate to continuous deviations of feedback from an intended target within a tracking task (Pulferer et al., 2023), paving the way to similar findings for deviations of a vehicle from the road within a car driving task. However, to the best of our knowledge, disentangling the superposition of all these processes within a car driving task has not yet been attempted.

In addition to the mentioned questions, previous research has not attempted to infer continuous signals such as car trajectories or steering motion from the EEG to the present day. While there have been approaches to regress ongoing continuous movement of both upper and lower limbs within a range of studies (Mondini et al., 2020; Tortora et al., 2020; Fu et al., 2022; Borra et al., 2023), utilizing both classical machine learning approaches as well as convolutional or recurrent neural networks, literature has not yet applied these methods to infer task-related signals during car driving. However, these findings could provide valuable information, both for vehicle safety in general and brain-computer interfaces in particular.

In the present study, we thus aimed to investigate the cortical activity within a simulated car driving task for both observed and executed driving, employing passive (steering along), proactive (unhindered steering), as well as reactive (corrective) steering conditions, comprising error-free and error-prone vehicle operation conditions. We simultaneously recorded EEG signals in pairs of participants within 13 sessions (26 participants in total), wherein each participant took on the role of Executor (person actively steering) and Observer (person merely observing) once. Additionally, both the steering wheel signal and the car position were recorded to enable time-locking to specific time points of interest such as maxima in steering wheel deflection or car deviation from the road. We hypothesized that different brain regions should reach synchrony at different time-locks depending on the specific processes they are tasked with. Further, we utilized a slight modification of EEGNet, a well-known convolutional neural network architecture in the context of EEG decoding, to infer both the steering signal and the current car position on-screen from the EEG; an endeavor not yet reported within literature so far. Within sensor space, time-frequency, directional connectivity, and source localization analyses, we overall aimed to identify distinct brain patterns relating to steering motion, visual processing, and erroneous feedback processing.

## 2 Methods

### 2.1 Participants

Within a total of 13 sessions in pairs of two, 26 able-bodied participants [age: 23.8 (mean) ± 2.2 (SD) years; age range: 20 to 28 years; 13 male, 13 female] underwent a car driving task employing varying vehicle operation conditions. Of all participants, each reported normal or corrected-to-normal vision, and 15 confirmed prior participation in an EEG study. We assessed 23 persons as right-handed, and three as left-handed, according to the Edinburgh Handedness Inventory (Oldfield, 1971). Each participant gave their written informed consent and received compensatory payment for their time. All measurements were conducted at the Graz University of Technology and approved by the local ethics committee.

### 2.2 Data acquisition

During each session of recordings, the EEG and electrooculogram (EOG) of two participants – each of which operated as an Observer and as an Executor once - were recorded simultaneously on 64 active electrodes per person (actiCAP, Brain Products GmbH, Gilching, Germany). The total setup corresponded to a 60-channel EEG according to a modified 10–10 system layout (Supplementary Figure S1), as well as a 4-channel EOG, both recorded at a sampling rate of 200 Hz. As in previous work (Martínez-Cagigal et al., 2020; Pulferer and Müller-Putz, 2022) six electrodes from frontopolar and temporal sites (Fp1, Fp2, FT9, FT10, TP9, TP10) were removed, and reallocated to two parietooccipital electrode positions for increased channel density above the cuneus and precuneus area (PPOh1, PPOh2; according to the 10–5 system), as well as four EOG locations (above and below the left eye and the outer canthi of both eyes). Ground and reference electrodes were placed at the electrode position Fpz and on the participants’ right mastoid, respectively.

All data were recorded and synchronized via lab streaming layer (https://github.com/sccn/labstreaminglayer). The presented main paradigm was created and displayed utilizing MATLAB 2017b (MathWorks Inc. United States); the eye artifact correction package we employed (Kobler et al., 2020) additionally utilized Psychtoolbox (Brainard, 1997; Pelli, 1997; Kleiner et al., 2007).

### 2.3 Experimental design

**Figure 1:**
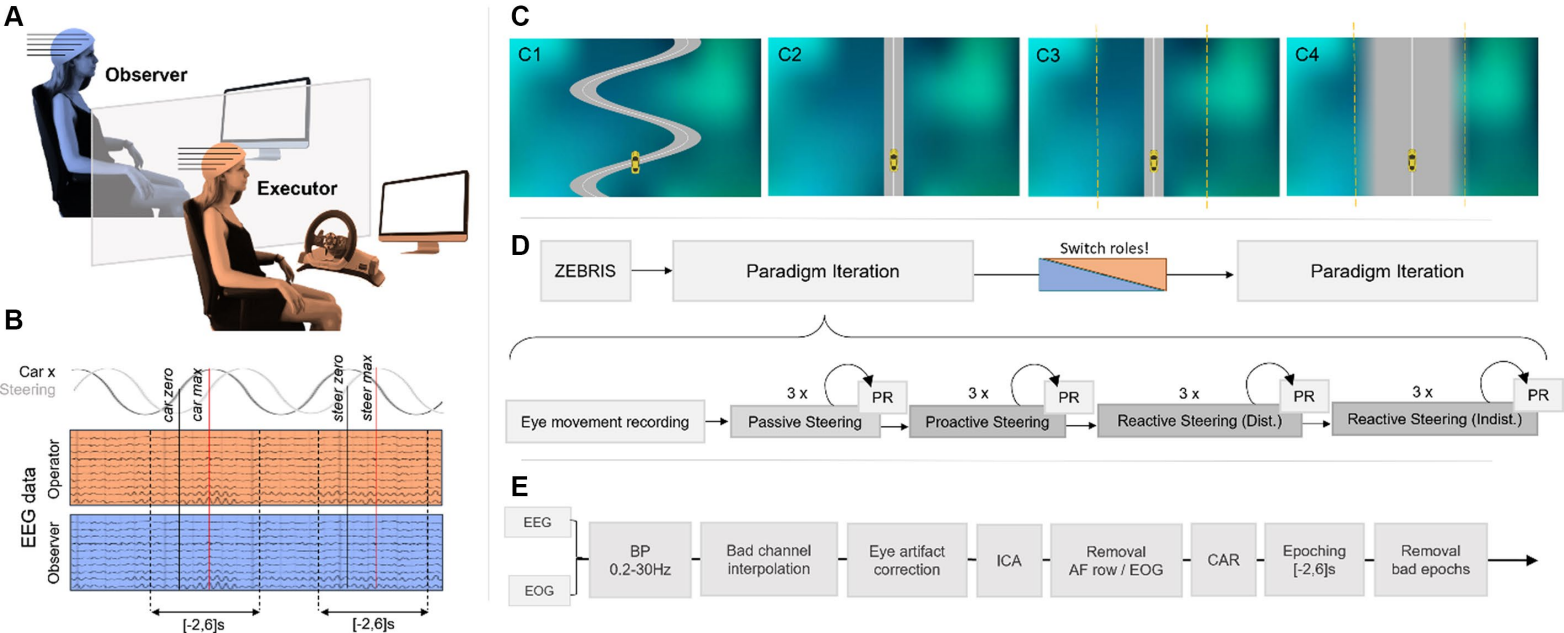
**(A)** Experimental setup. Both participants observed the driving performance on their screen (no visual contact with each other) as the Executor steered the car with an XBox driving wheel. **(B)** Epoching scheme. Executor and Observer EEG data were time-locked within [−2,6]s of zeros in the horizontal car position and the steering signal, respectively. **(C)** Car-driving conditions. A gray road with a white centerline on a shaded background, as well as a yellow car, were displayed for all conditions. The car remained on a narrow winding road by itself during the *Passive Steering* condition **(C1)**; the Executor had full steering control during the subsequent *Proactive Steering* runs on a straight, narrow, and distinct road **(C2)**. During the *Reactive Steering* conditions, participants faced a narrow and distinct road (**C3**; *Distinct*) and a wide and blurred road (**C4**; *Indistinct*). During *Reactive Steering* conditions, steering control was revoked within a fixed margin around the road (marked with a yellow dashed line here) to cause unintended vehicle behavior and provoke erroneous feedback processing. **(D)** Recording pipeline. Electrode positions were recorded for both participants before the first paradigm iteration started, consisting of eye movement recordings and a run-through of all four car-driving conditions in three runs each with performance rating (PR) questionnaires after each run. **(E)** Processing pipeline.

Before each session, we assigned each of the two participants an initial role at random, corresponding to either Executor or Observer. The setup involving two participants at the same time was chosen as previous literature reported considerable differences between executed and observed erroneous action in the processing of discrete error stimuli (van Schie et al., 2004). The participant acting as Executor then took a seat in front of an Xbox Wireless Racing Wheel (Microsoft, 2006/2007; sans foot pedals) inside our measurement box, while the Observer was positioned outside (Figure 1A). This spatial separation prevented direct visual contact between the two participants; however, due to the noise caused by the steering wheel’s mechanics, we decided against closing the door to the measurement box to establish identical auditory paradigm conditions for Executor and Observer. Both participants faced their computer screens at a comfortable distance (~1 m).

The whole measurement session then consisted of two identical iterations through our paradigms. In detail, for each of the iterations, we first presented the participants with a run unrelated to the main hypothesis (6 min), designed to capture the rotational movement of the corneo-retinal dipole. This run specifically recorded vertical and horizontal eye movement, blinks, and resting state data (open-eyed), cued by a moving, pulsating, or static target on-screen, to create an eye artifact removal model for offline artifact correction (Kobler et al., 2020). No interaction with the steering wheel occurred during this first run. Afterward, the car-driving paradigms commenced, consisting of four different vehicle operation conditions labeled *Passive Steering, Proactive Steering, Reactive Steering (Distinct),* and *Reactive Steering (Indistinct)*. Each condition was presented in three separate runs (3.5 min each), adding up to around 15 min of recordings per condition including instructions, and totaling to approximately 1 h of recordings for one paradigm iteration (Figure 1D). To capture the subjective perception of the Executor’s steering performance per condition and run, we additionally asked both participants to rate the perceived performance on a visual analog scale ranging continuously from 0 (bad performance) to 1 (excellent performance).

After the first paradigm iteration, the participants took a short break before the roles were reversed and the second paradigm iteration started up. Specifically, the person previously acting as Executor left the box and switched positions with the former Observer to henceforth observe from the outside as the new Observer. Likewise, the former Observer took their place at the steering wheel, acting as the new Executor for the rest of the measurement. This switch allowed us to capture both observation and execution data of each participant. The measurement commenced again with a second eye movement recording run, followed by the four car-driving paradigm conditions.

#### 2.3.1 Driving simulation

For all car-driving conditions, the paradigms comprised a bird-view environment depicting a gray road with a white center line, as well as a yellow car to operate (Figure 1C). The car’s initial position corresponded to the center of the screen in the horizontal axis and approximately a fourth of the screen’s height from the bottom up in the vertical axis, henceforth considered the origin of our coordinate system when describing the car’s position on-screen). Additionally, we set a randomized non-zero initial horizontal velocity to enforce a necessity of steering within the *Proactive Steering* ( $v_{x,0}=\pm 5px/s$ ) and both *Reactive Steering* [ $v_{x,0}\in \left(-300,300\right)px/s$ ] conditions, while the vertical velocity was fixed to zero throughout the experiment. As such, a sense of forward motion was exclusively enforced by moving the shaded background downwards at a constant speed. Starting from these initial settings, the car’s position was updated at 50 Hz by integrating the current position from the previous velocity and position, and directly adding the current steering input weighted by a fixed weighting factor ( wf ) per condition; the current velocity was then in turn differentiated from the new kinematics. The fixed weighting factor was set higher for the *Reactive Steering* conditions ( wf=30 ) compared to the *Proactive Steering* condition ( wf=5 ) to raise the level of steering difficulty. As the current work aimed to analyze the cortical response to recurring lane deviations rather than the overall driving experience, the simulation was kept as minimalistic as possible.

#### 2.3.2 Car-driving conditions

For each of the car-driving conditions, we instructed both participants to focus their attention on the moving car while the Executor steered with both hands according to the instructions delivered directly before the commencement of the different conditions. We additionally instructed the participants that steering errors causing a deviation from the road should be perceived as more drastic than errors that only caused a mere deviation from the white centerline while remaining on the road, thus introducing different levels of urgency to the erroneous feedback processing.

##### 2.3.2.1 Passive steering

During the runs of the *Passive Steering* condition (Figure 1C1), the participants faced a winding road spanning approximately half of the screen’s width. In contrast to the other presented main paradigms, the car stayed exactly on the road by itself, moving along the turns at a frequency of 0.25 Hz (i.e., the duration between maximum displacement to the right and consecutively to the left corresponded to 2 s). Both participants were informed about the fake nature of the feedback beforehand; we instructed the Executor to steer along as if they were in control of the car, even though they were not.

We set the movement along the road at an exact frequency without jittering to observe the influence of periodic stimulus movement on the brain signals. In addition, the *Passive Steering* condition (i.e., merely steering along to the self-moving car) served to disentangle cortical activity related to generic (passive) and self-reliant (proactive) motor output.

##### 2.3.2.2 Proactive steering

During *Proactive Steering* condition runs (Figure 1C2), the Executor wielded full steering control and was instructed to attempt to always stay on the white centerline of a straight and narrow road. The steering settings were chosen such that all participants could comfortably reach this goal. The *Proactive Steering* condition was designed to provide information on how the brain operates during successful fine-tuned feedback control.

##### 2.3.2.3 Reactive steering (distinct)

As in the *Proactive Steering* runs, the *Reactive Steering (Distinct)* runs (Figure 1C3) depicted a straight and narrow road again, and the Executor was similarly instructed to attempt to always stay on the white centerline. In contrast to the *Proactive Steering* scenario however, the steering control for the *Reactive Steering (Distinct)* runs was momentarily revoked whenever the car’s deviation from the centerline fell below one-fifth of the screen’s width. In practice, this was achieved by removing the steering input when integrating the current velocity - and thus forcing the system to instead depend on the prior kinematics only - whenever the car crossed the threshold. As a result, the Executor had no control within this margin around the road, and the car would instead cross over the centerline unhindered until it exited the margin on the other side again. At this point, the Executor regained control and could steer back toward the road once again. To prevent excessive steering off the road, we instructed the Executors to attempt to return to the centerline swiftly after regaining control. We employed this specific control mechanism to enforce continuous erroneous feedback processing in the form of lane deviations. In our hypothesis, the limited control offered in this condition should cause considerable engagement of feedback monitoring and error processing networks in the brain.

##### 2.3.2.4 Reactive steering (indistinct)

During *Reactive Steering (Indistinct)* runs (Figure 1C4), we finally aimed to investigate differences in cortical modulations with the perceived importance of an error. To this end, participants faced the same limited control scenario as during the *Reactive Steering (Distinct)* condition (the margin of revoked steering control around the road remained unchanged). However, instead of the narrow and distinct road shown previously, the *Reactive Steering (Indistinct)* runs instead depicted a broad road with blurred edges. The premise that getting off the white centerline already constituted a steering error, but getting off the road should be perceived as even more drastic, remained intact. However, we hypothesized that the larger area of the road, as well as its vague boundaries, should lead to a different perception of urgency with faulty steering, and thus only provoke minor engagement of related cortical networks compared to *Reactive Steering (Distinct)* runs.

### 2.4 Data processing

As previous research reported contributions of various frequency bands to error processing in the brain (Yordanova et al., 2004; Koelewijn et al., 2008; Carp and Compton, 2009), we chose to bandpass filter both EEG and EOG signals at 0.2-30 Hz (Butterworth, 10th order). Subsequently, we identified and spherically interpolated noisy channels via visual inspection, abnormal kurtosis, and abnormal joint probability (exceeding five standard deviations of the mean for both measures). We used EEG and EOG data of the eye movement recordings to train an eye artifact correction model to correct the hypothesis-related EEG data of the main paradigm conditions for rotational movements of the corneo-retinal dipole (Kobler et al., 2020), followed by ICA [FastICA algorithm (Hyvärinen, 1999), https://research.ics.aalto.fi/ica/fastica/index.shtml] in the case of a persisting influence of ocular movement on the EEG. To this end, the independent components were first automatically labeled using the ICLabel classifier available in EEGLab (Pion-Tonachini et al., 2019). Components attributed to eye, heart, or muscle artifacts with a classification accuracy of at least 90% were then automatically removed. The scalp distribution of the ICA weights, as well as the time series of the components, were then visually inspected to exclude remaining eye, heart, or muscle artifacts. The EOG and anterior frontal row electrodes were then excluded from further analysis, leading to a remaining total of 55 channels to consider which were then re-referenced to their common average. The whole processing approach is depicted in Figure 1E.

In terms of non-biological signals, the car’s x-position signal was analyzed as recorded without further processing. To alleviate jitters in the recorded steering wheel signal, we smoothed the data with a Savitzky–Golay filter utilizing second-order polynomials to fit windows of 351 samples (1.75 s) (Savitzky and Golay, 1964).

### 2.5 Data analysis

To obtain an understanding of the differences in cognitive processing between the four car-driving paradigm conditions, as well as differences between execution and observation of the task, we analyzed the recorded data both in sensor space and in source space. Due to the lack of normality in the distribution of single evaluation metrics, non-parametric tests were selected to assess significant differences between populations. To this end, significant differences between pairs of populations (e.g., Executor vs. Observer) were evaluated using a Wilcoxon signed rank test; for comparisons between more than two populations (e.g., the four car-driving conditions) were assess with a Friedman-Nemenyi test – a many-sample extension of the Wilcoxon test (Zimmerman and Zumbo, 1993). Both tests were followed by subsequent False Discovery Rate (FDR) correction for multiple comparisons.

#### 2.5.1 EEG data

To identify different brain regions tasked with individual subprocesses of car driving, we chose to analyze two different time-locks of interest within this work: (a) a time-lock to zero-crossings in the car’s x position (henceforth termed *car zero*), and (b) a time-lock to zero-crossings in the steering wheel signal (henceforth *steer zero*). Considering the periodic left-and-right movement of the car, the *car zero* time-lock served to trace visual/feedback processing, while the *steer zero* time-lock should identify movement-related activity in the brain. For both time-locks, we epoched the continuous EEG data to slices within [−2,6]s. As a last step, we rejected bad epochs based on visual inspection, thresholding (exceeding ±100 μV), abnormal kurtosis, and abnormal joint probability (exceeding five standard deviations of the mean for both measures).

As the steering behavior differed considerably between the displayed car-driving conditions, comparing the grand average signals throughout the epoch length across conditions was considered as meaningless. Instead, we chose to additionally track consecutive extrema for each of the two time locks, i.e., the succeeding maxima in horizontal car deviation from the origin (*car max*), and in steering wheel deflection (*steer max*). To compare between conditions then, only the topographical maps at the four time points *car zero/max* and *steer zero/max* were considered.

To utilize the full epoch information nonetheless, we selected the channel displaying the largest signal amplitude within those four time points per condition, corresponding to either the electrode position FCz, Cz, or Pz. This selection was made to display the recorded cortical activity meaningfully yet compactly per trial, as in principle, these maps would have been possible for all EEG channels. For this singular channel, we then considered all trials per participant and sorted them in ascending order according to their latency between zero-crossings (i.e., *car*/*steer zero*) and consecutive extremum (i.e., *car/steer max*). As the number of trials varied between participants, we then spherically interpolated the sortedtrials×timepoints matrix at the specific EEG channel per participant along the trial dimension to obtain 100 interpolated ‘pseudo trials’ for each participant [i.e., a ( 100×timepoints ) matrix], in line with similar approaches by Burle et al. (2008). A matching procedure was used to spherically interpolate the corresponding latencies. The resulting sorted and interpolated trials for the selected EEG channel, as well as the latencies, were then grand averaged across all participants to display changes in EEG activity with steering activity and car position.

#### 2.5.2 Frequency analysis

**Figure 2:**
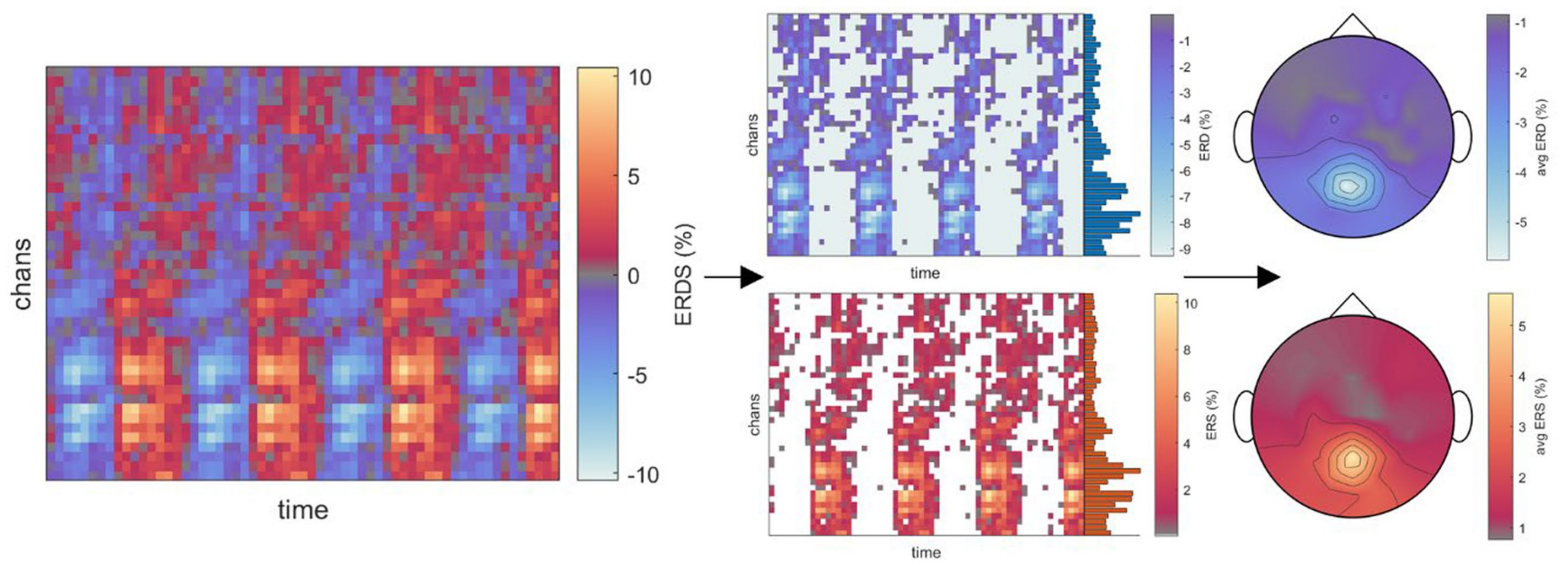
Visualization of the time-frequency analysis procedure. Due to the periodic stimulus (car) movement, repeated instances of ERD/ERS arose in recurring brain regions within one epoch (left panel; example from Executors’ data, *car zero* time-lock, *Passive Steering* condition, alpha frequency band). As no specific time points stood out, ERD and ERS were instead separated by masking values of opposite signs across the epoch (middle panels). The resulting, strictly negative (ERD) or positive (ERS) values were then averaged across time to produce topographical maps displaying the overall ERD/ERS activity per channel throughout the epoch.

To analyze the time-frequency behavior in each role and condition, we calculated the one-sided Short-Time Fourier Transform (STFT) of the 55-channel EEG on each 8 s-long single trial (i.e., 1,601 time points at 200 Hz), using a 200-sample Hanning window at 175 samples of overlap in a 256-point Fast Fourier Transform (FFT). Spectrograms displaying a signal power beyond ±3 standard deviations from the mean were excluded from further analysis, corresponding to on average 4.3% (STD 9.0%) of trials across all conditions. For each role, condition, and participant, the resulting single-trial spectrograms were then averaged across trials and within the frequency ranges corresponding to delta (1-4 Hz), theta (4-8 Hz), alpha (8-13 Hz), and beta (13-30 Hz) bands. We then calculated the event-related desynchronization/synchronization (ERD/ERS) as initially described by Pfurtscheller and Aranibar (1977) and Pfurtscheller (1992) using the whole epoch as a baseline. As steering behavior and visual feedback varied considerably across time between conditions, making a comparison of time points meaningless, we chose to mask the positive (ERS) and negative (ERD) values in the channels×time points map obtained for each frequency band of interest, respectively. The masked maps were then averaged across all time points to obtain a topographical map corresponding to the time-averaged ERD/ERS arising throughout the epoch, made possible by the recurring desynchronization/synchronization of the same scalp regions. The procedure is exemplified in Figure 2 for the alpha band in the Executor’s *Passive Steering* condition within the *car zero* time-lock.

#### 2.5.3 Directional connectivity analysis

In line with Granger and Wiener’s description of causal relation between time series (Granger, 1969), we utilized multivariate autoregressive (MVAR) models to analyze the causal connections between the single EEG channels. A causal connection may accordingly be assumed if knowledge of past values of one time series improves predictions of another time series. These MVAR models can be generalized to.


(1) $$y\left(n\right)=\overset{p}{\underset{k=1}{\sum }}A_{k}y\left(n-k\right)+\epsilon \left(n\right)\text{,}$$

where $y\left(n\right)\in R^{\left(M\times 1\right)}$ denotes a multivariate time series at time point n (in this case, the 55-channel EEG), $A_{k}\in R^{\left(M\times M\right)}$ the static coefficient matrices for time lags k∈N up to model order p∈N , $y\left(n-k\right)\in R^{\left(M\times 1\right)}$ the regressor vector in the form of the multivariate time series at lag k , and $\epsilon \left(n\right)\in R^{\left(M\times 1\right)}$ the innovation process vector in the form of zero-mean white noise. In matrix form, the stacked coefficient matrices $A=\left[A_{1},\ldots ,A_{p}\right]\in R^{\left(M\times Mp\right)}$ can be estimated via Least Squares approach; in this work, we opted to use the Arfit module employing a stepwise least squares algorithm (Schneider and Neumaier, 2001; Schlögl, 2006). As model performance crucially depends on the selected model order p , i.e., on the number of past time points considered for prediction, we selected the optimum model order $p_{\mathrm{opti}}$ as the model order $p\in \left[1,50\right]$ minimizing Schwarz’s Bayesian Criterion (Schwarz, 1978) implemented in the Arfit module.

As the contribution of each individual time lag to the model’s performance provided limited information in our case, we rather chose to investigate the oscillatory content in the frequency domain as obtained by Fourier-transforming the relationship of Eq. (1) (Saito and Harashima, 1981; Akaike, 1998; Baccalá et al., 1998). Using the transformation property of the lag operator, $F\left\{y\left(n-k\right)\right\}=\exp\left(-2\mathrm{\pi ikNf}\right)y\left(f\right)$ , with $y\left(f\right)$ the Fourier transform of $y\left(n\right)$ , the frequency representation of $y\left(f\right)={\left[I-\overset{p}{\underset{k=1}{\sum }}A_{k}\;\exp\;\left(-2\mathrm{\pi ikNf}\right)\;\right]}^{-1}E\left(f\right)=H\left(f\right)E\left(f\right)$ can be obtained, with $E\left(f\right)=F\left\{\epsilon \left(n\right)\right\}$ , and $H\left(f\right)$ the transfer matrix projecting the frequency content of our zero-mean white noise to the frequency content observed in our multivariate time series. Various connectivity measures utilize $H\left(f\right)\in R^{M\times M}$ to infer directional connectivity within the M time series [see for instance (Faes et al., 2013)]; for this work, we employed the directed transfer function (DTF) (Kamiński and Blinowska, 1991):


(2) $$DTF_{ij}\left(f\right)=\frac{\left|H_{ij}\left(f\right)\right|}{\sqrt{\sum_{m=1}^{M}{\left|H_{im}\left(f\right)\right|}^{2}}}\text{.}$$

Each entry ij of the asymmetric matrix $DTF\left(f\right)\in R^{M\times M}$ then describes both direct and indirect contributions of a driving time series j to a target time series i. Per definition, the total contribution of all driving channels to each single target channel of $DTF{\left(f\right)}^{2}$ is normalized, i.e., $\overset{M}{\underset{k=1}{\sum }}DTF_{ik}{\left(f\right)}^{2}=1$ ; in contrast, summing the contributions to all possible target channels in one single driving channel, i.e., $\overset{M}{\underset{k=1}{\sum }}DTF_{kj}{\left(f\right)}^{2}$ , yields a measure for the total information outflow of each driving channel.

To estimate the DTF matrix, the eMVAR toolbox by Faes et al. (2013) was used. To remove inter-participant variations within the EEG unrelated to the relevant information processing, we standardized the subject averages before model fitting. For each role (Observer, Executor) and condition [*Passive Steering, Proactive Steering, Reactive Steering* (*Distinct/Indistinct*)], we then considered the averaged epochs of EEG data in all participants as our multivariate time series of interest, generating a regressor matrix $Y\in R^{M\times NS}$ with S=26 participant averages, N=1601 time points, and M=55 EEG channels. Statistical significance of the resulting DTF matrix within each role and condition was evaluated using 50 surrogate signals, as previously employed in other work (Faes et al., 2010; Kostoglou and Müller-Putz, 2021; Wimmer et al., 2022). For this purpose, artificial time series (surrogates) were generated by Fourier-transforming the initial multivariate time series, shuffling their phases, and then back-transforming to the time domain. For each surrogate, now void of any causal relation regarding the frequency content, an MVAR model was fitted to the previously found optimum model order $p_{\mathrm{opti}}$ , and the DTF matrix was calculated. The threshold for statistical significance in each target-driver pair was then set to the 95th percentile of those 50 surrogate DTF results, below which any results were set to zero, indicating no significant directional connectivity within that specific driver-to-target combination.

#### 2.5.4 Source space analysis

In addition to the explicit analysis of the recorded EEG data, we back-projected the signals to the cortex using Brainstorm [Version: 16-Mar-2023; Tadel et al. (2011)]. To this end, we recorded the exact electrode positions for all participants before each session of recordings (ELPOS, Zebris Medical GmbH, Germany) and co-registered the ICBM152 boundary element model [BEM; Kybic et al. (2006)] to the specific electrode positions. To alleviate deviations of the individualized electrode positions from the template head model due to differences in head geometry, we projected the positions on the model’s surface. Further, we adjusted the standard values for cortex, skull, and scalp conductivities in the BEM to 1, 0.008, and 1. The required noise covariance matrix for calculating forward [OpenMEEG, Gramfort et al. (2010)] and inverse solutions [sLORETA, Pascual-Marqui (2002)] was estimated from a resting state condition included in the eye movement recording runs (Srisrisawang and Müller-Putz, 2022) and regularized by adding an identity matrix scaled to 10% of the largest eigenvalue. To investigate the valence of the source signals rather than the signals’ modulus, we constrained the model to only consider one dipole source per voxel oriented perpendicular to the cortex. This led to a total of 15,000 source dipoles across the whole cortex. After projecting the averaged signals per participant to the source space, we thus obtained grand average source maps for both Observer and Executor in all four car-driving conditions.

To further investigate relevant brain regions within each role and condition, we downsampled the full cortical maps to 68 regions of interest as defined within the Desikan-Killiany atlas (Desikan et al., 2006) spanning the whole cortex.

### 2.6 Regressing car x position and steering wheel signal

To gain a general understanding of how both the car’s position (for both roles) and the steering process (Executors only) are represented in the brain, and to specifically test which of both non-biological signals can more accurately be inferred from the EEG, we chose to slightly modify the EEGNet architecture for regression on our dataset (Lawhern et al., 2018). The architecture, a three-layer convolutional neural network, was chosen as EEGNet reportedly provided high performance within a range of tasks in brain signal decoding (Lawhern et al., 2018). As the goal of this approach was predominantly to provide first results for regressing car positions and steering information from the brain signals, other undoubtedly equally suitable deep learning architectures, such as deep believe networks (An et al., 2014), recurrent neural networks (Tortora et al., 2020), or even transformer models (Song et al., 2023), have not been investigated in the current work.

For each of the four conditions, a 1 s-window (corresponding to T=200 samples) was slid through the continuous data of each run with a stride of 15 samples (75 ms) to obtain a total of 7,920 frames per condition. To prevent possible contributions of muscular activity at EEG channels near the neck to the regression performance, all EEG channels comprising the outermost circle were excluded, yielding windowed data in C=43 EEG channels of the form $X\in R^{7920\times 43\times 200}$ . To retain a causal relationship between training and testing data, we then split the frames into six folds, the last of which we retained for testing. Both training and testing data were standardized with respect to the training dataset.

The network architecture processed batches of inputs of the size C×T in three consecutive layers. In the first layer, inputs were zero-padded with 32 samples before and after the input in the time dimension, followed by temporal convolution with a kernel size of (1,64) which corresponded to a high pass cut-off at approximately 3 Hz. Within the second layer, we applied depthwise convolution across all available channels [kernel size (43,1)], followed by average pooling with a kernel size of (1,4). A third (final) convolutional layer with kernel size (1,32) further extracted temporal features, followed by a second instance of average pooling with a kernel size of (1,8). Finally, the resulting features were flattened and passed through a fully connected layer returning one real-valued output corresponding to the final prediction of the regression model. Each convolutional layer was followed by a batch normalization layer to accelerate convergence (Ioffe and Szegedy, 2015), as well as a dropout layer (0.25 dropout rate each) to impede overfitting. Exponential Linear Units were used for activation functions (Clevert et al., 2015).

We generated separate models utilizing the same hyperparameters to regress the car’s x position and the steering wheel signal for each condition in the Executors’ data. In detail, the models were trained for 100 epochs at a learning rate of 10–4 with a batch size of 256. We used the Adam optimization algorithm implemented in PyTorch to optimize the model parameters with respect to the mean squared error loss between the prediction and dependent variable (car position or steering signal).

To analyze the importance of each input channel to the model’s performance, we further calculated saliency maps on the testing data. To this end, the highly non-linear mapping between a two-dimensional input frame I (i.e., the C×T frames of windowed EEG data) and the scalar regression output $S\left(I\right)$ (predictions) through the neural network is linearly approximated in a first-order Taylor expansion around a given input frame $I_{0}$ as


(3) $$S\left(I\right)\approx w^{T}I+b={\left({\left(\frac{\partial S}{\partial I}\right)|}_{I_{0}}\right)}^{T}I+b\text{.}$$

In practice, for each (flattened) given input frame of the test set, the model weights $w\in R^{\left(43*200\right)\times 1}$ are found via backpropagation, yielding a final saliency map $M\in R^{\left(43\times 200\right)}$ . Each entry of M then corresponds to the absolute of the matching entry of w (Simonyan et al., 2013) and indicates the contribution of a specific channel and time-point to the network prediction. A topographical feature importance map is then obtained by summing all entries along the time dimension.

The network was implemented using PyTorch (https://github.com/pytorch/pytorch); feature importance analyses were conducted with the Captum library (https://github.com/pytorch/captum).

## 3 Results

In the following paragraphs, we present grand average EEG results as obtained by time-locking the EEG signals to zero-crossings in the steering signal and zero-crossings in the car x position, respectively. This was done to identify specific brain regions phase-locked to either of these non-biological signals, i.e., regions involved in executive control via time-locking to the steering signal, and regions relating to both visual and feedback processing via time-locking to the car’s position on-screen. Concerning the independent component analysis, we excluded on average 3.3 (STD 2.6) components for all participants and conditions.

### 3.1 Differences in steering behavior between conditions

**Figure 3:**
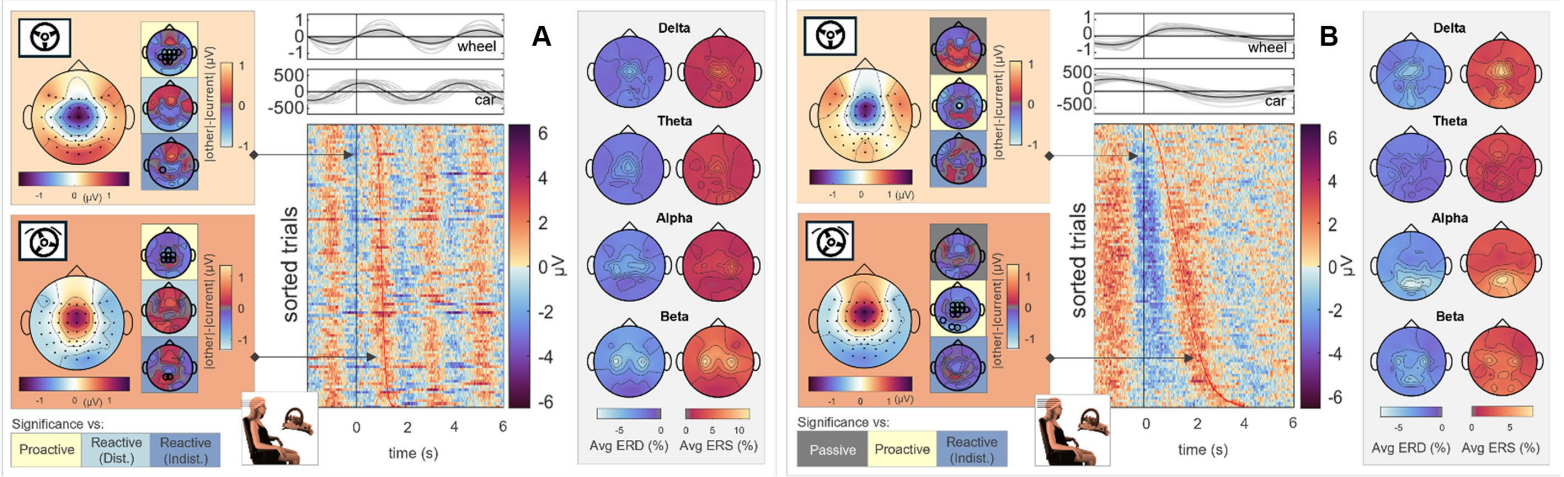
Execution, *steer zero* time-lock. Grand average topographical maps at the time-lock (*steer zero*, light orange) and maximum steering wheel deflection (*steer max*, dark orange). Pairwise significant differences to the other conditions are shown as black circles in the adjacent topographical maps (Friedman-Nemenyi test, FDR-corrected at 0.05). Grand-average sorted and interpolated trials at electrode position FCz, time-locked to zeros in the steering wheel signal (*steer zero*, black line). Time points of maximum steering wheel deflection (*steer max*) per trial are indicated with a red line; participant averages (grey) of the steering wheel signal and car x position with their corresponding grand averages (black) are shown on top. ERD/ERS patterns, time-averaged across the epoch length as described, in four frequency bands of interest. All results are shown for **(A)** *Passive Steering* and **(B)** *Reactive Steering (Dist.)* conditions.

To analyze differences in steering behavior, we time-locked the EEG signals to zero-crossings of the steering wheel’s signal for all four conditions. As only the Executor performed the steering, we omitted the Observer’s data from further analysis utilizing this specific time-lock. Results for the *Proactive Steering* and the *Reactive Steering (Indist.)* conditions are provided in the Supplementary Figures S2, S4, upper panels. A first analysis of the scalp topographies at the time-lock (i.e., *steer zero*) and the subsequent time points maximum steering wheel deviation (*steer max*) revealed comparable frontocentral positivities across all conditions, disregarding the corresponding type of control (*Passive, Proactive,* or *Reactive Steering*). The results for the *Passive Steering* and *Reactive Steering (Dist.)* conditions are shown in the lefthand side topographical maps of Figures 3A,B, respectively. Significant differences only arose in comparisons with the *Proactive Steering* condition, while a slight frontal shift in activity occurred for all other conditions when compared to the *Passive Steering* condition. The full time courses of the grand averaged EEG signals in all conditions within the *steer zero* time-lock for Execution and Observation are provided in the Supplementary Figures S8, S9.

Considering the focal frontocentral activity across conditions, we selected the EEG channel at electrode position FCz for sorting the trials according to their steering latencies. The resulting grand-averaged sorted and interpolated trials of the *Passive Steering* and *Reactive Steering (Dist.)* conditions are shown in the image plots of Figures 3A,B, respectively. We observed a clear modulation of brain activity recorded at position FCz with the steering activity, with positivities in the brain signals phase-locked to instances of maximum steering wheel deflection (Figures 3A,B, red line in the image plots). For the *Passive Steering* condition, which followed a pre-defined periodicity, the patterns visibly repeated with the steering motion throughout the epoch length, whereas the slower (self-paced) steering within the *Reactive Steering (Dist.)* condition occurred at a larger time frame. Interestingly, we observed very distinct ERD/ERS in the *Passive Steering* condition (Figure 3A, topographical maps in grey boxes). Delta and theta bands exhibited weak central scalp topographies, whereas alpha and specifically beta bands showed focal activity for EEG channels at electrode positions C3 and C4. Similar, though more frontal delta ERD and ERS topographies arose for the *Reactive Steering* conditions (Figure 3B, topographical maps in grey boxes), whereas no clear patterns were observed for the theta frequency band. We also noted a clear shift in alpha activity to occipital regions for both ERD and ERS maps, as well as additional occipital activity in the beta band.

**Figure 4:**
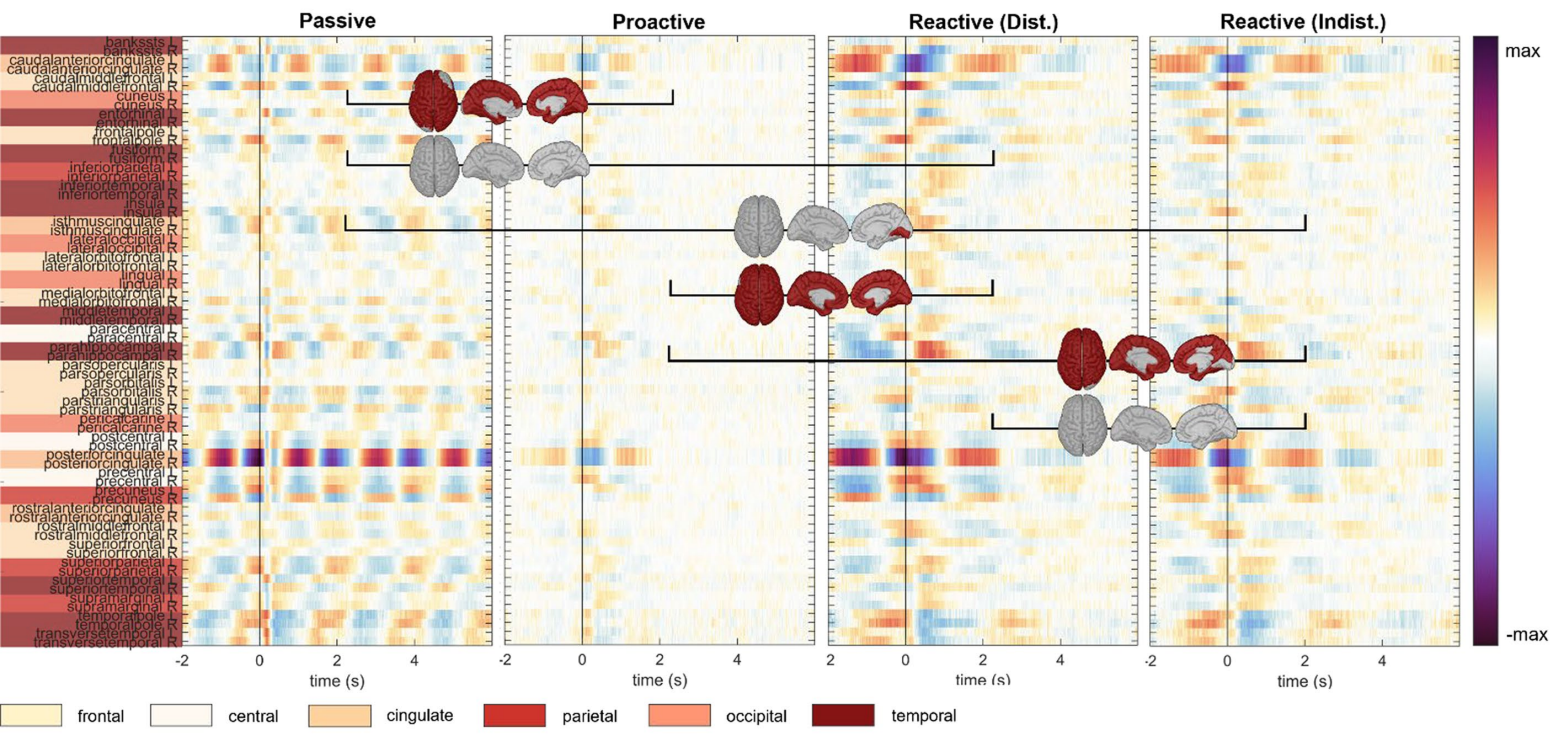
Execution, *steer zero* time-lock. Grand-average cortical activity at 68 brain regions defined within the Desikan-Killiany atlas for *Passive, Proactive, and Reactive Steering (Dist./Indist.)* conditions (f.l.t.r.). Pairwise significant differences (Friedman-Nemenyi test, FDR-corrected at 0.01) are shown as dark red patches on the cortical maps.

An analysis of the cortical regions of interest comprising the Desikan-Killiany atlas is depicted in Figure 4; cortical maps between the single condition results indicate regions of significant pairwise difference at a significance level of 0.05 (dark red patches, FDR corrected for multiple comparisons). The cortical activity within the *Proactive Steering* condition (second panel from the left) showed strong attenuation and proved significantly different from all other conditions for most regions of interest; in contrast, no overall significant differences were seen for pairwise comparisons in other conditions. Interestingly, strongest activations arose in the bilateral caudal anterior (cACC) and posterior cingulate cortices (PCCs), parahippocampal and precentral gyri, and precuneus area across all conditions, with additional strong unilateral engagement of prefrontal cortex areas.

**Figure 5:**
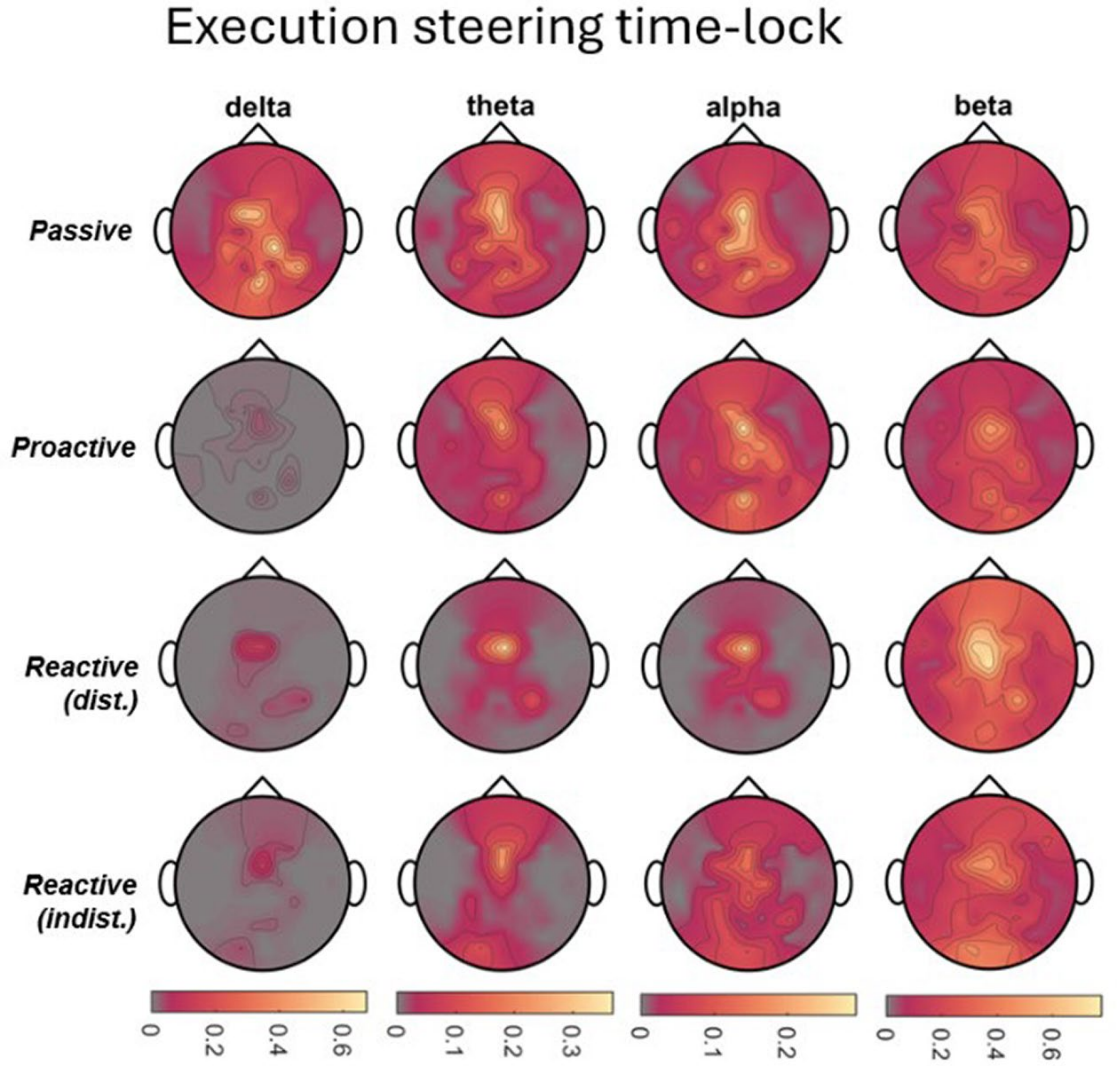
*Steer zero* time-lock. Total information outflow per channel as obtained from the Directed Transfer Function (DTF) for *Passive, Proactive,* and *Reactive Steering (Dist./Indist.)* conditions (f.t.t.b.) in four frequency bands of interest for Execution.

To analyze directional connectivity within all EEG channels, we utilized the standardized participant averages in the *steer zero* time-lock, yielding a multivariate time series matrix $X\in R^{M\times N\times S}$ , with M=55 channels, N=1601 time points [(−2,6)s epoch], and S=26 participants. The optimum model orders per condition and role were exclusively found to be $p_{\mathrm{opti}}\in \left\{21,22\right\}\text{.}$ In line with the time-frequency analysis, we then averaged the outflow across the four frequency bands of interest (delta to beta). Topographical maps displaying the total information outflow per band in the different paradigm conditions are shown in Figure 5. In detail, we observed large-scale information outflow at central and parietal regions during the *Passive Steering* condition across all frequency bands. In contrast, these regions condense increasingly toward frontocentral sites as the frequency is lowered; specifically in the delta band, little significant information outflow remains apart from regions at approximately electrode position FCz. Notably, an increase in information outflow from these frontocentral sites within the beta band arises in the *Reactive Steering* conditions.

### 3.2 Differences in feedback processing between conditions

To assess differences in visual and feedback processing between conditions and roles, we additionally time-locked the EEG signals to zero-crossings of the car’s x position for all four conditions. In the case of the *Passive Steering* trials, this merely implied time-locking to every instance where the car passed through the screen’s center, while for *Proactive* and *Reactive Steering* conditions, it additionally corresponded to the car’s maximum deviation from the straight road. In contrast to the steering time-lock employed before, we here considered both Observer and the Executor data, as the visual input related to the car position time-lock comparably affected both roles.

**Figure 6:**
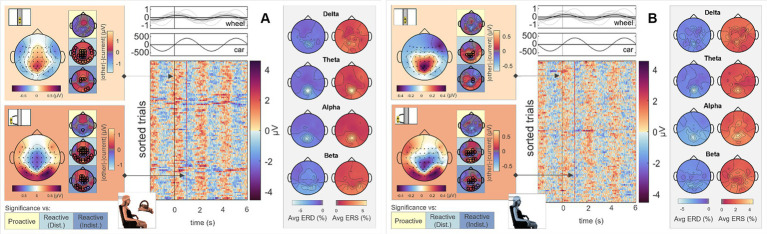
*Passive Steering*, *car zero* time-lock. Grand average topographical maps at the time-lock (*car zero*, light orange) and maximum car deviation from the road (*car max*, dark orange). Pairwise significant differences to the other conditions are shown as black circles in the adjacent topographical maps (Friedman-Nemenyi test, FDR-corrected at 0.05). Grand-average sorted and interpolated trials at electrode position Pz, time-locked to zeros in the car x position (*car zero*, black line). Time points of maximum car deviation from the road (*car max*) per trial are indicated with a red line; participant averages (grey) of the steering wheel signal and car x position with their corresponding grand averages (black) are shown on top. ERD/ERS patterns, time-averaged across the epoch length as described, in four frequency bands of interest. All results are shown for **(A)** Execution and **(B)** Observation.

To analyze cortical responses in Observers and Executors to a moving stimulus sans error connotation, we first analyzed the scalp topographies during zero crossings (*car zero*) and maximum deviation from the screen’s center (*car max*) in the *Passive Steering* condition. The results are displayed in Figure 6A (Executor) and B (Observer). As can be seen, the topographical maps for both time points largely coincide for both roles, with slightly increased central activity for the Executors and overall attenuated signal amplitudes in the Observers (see scale). Statistical analyses revealed significant differences between this *Passive Steering* condition and both *Reactive Steering* conditions (small topographical maps; channels displaying significant differences are mapped as black circles) at a significance level of 0.05 (FDR corrected). The full time courses of the grand averaged EEG signals in all conditions within the *car zero* time-lock for Execution and Observation are provided in the Supplementary Figures S6, S7.

To analyze the relation between brain activity and car position, we sorted and interpolated all EEG trials at electrode position Pz (highest activity as observed in the topographical maps); the results are shown in the image plots of Figures 6A,B. Interestingly, we observed clear modulations of the parietal brain activity with the phase of the car signal, i.e., positivities in the EEG coinciding with the car’s center position, negativities coinciding with the car’s maximum left/right deviation from the center. In contrast to the steering time-lock, neither ERD nor ERS maps show similar C3/C4 activity in the alpha and beta bands. However, similar ERD/ERS patterns arose between Observers and Executors, with high parietal theta and alpha activity.

**Figure 7:**
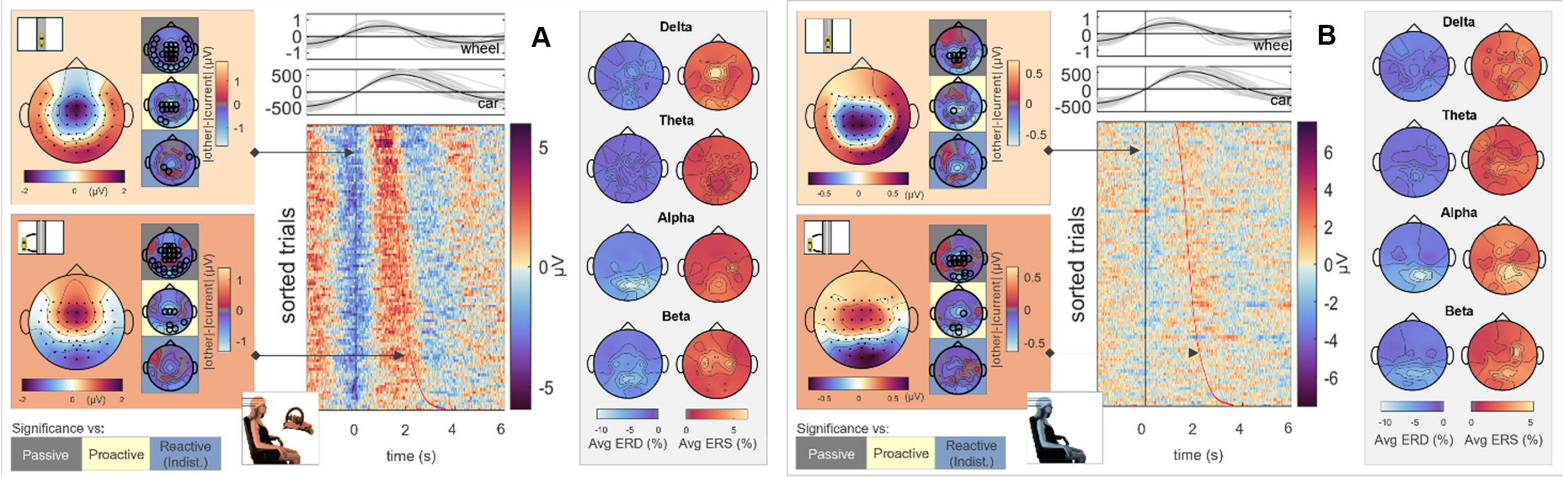
*Reactive Steering (Dist.), car zero* time-lock. Grand average topographical maps at the time-lock (*car zero*, light orange) and maximum car deviation from the road (*car max*, dark orange). Pairwise significant differences to the other conditions are shown as black circles in the adjacent topographical maps (Friedman-Nemenyi test, FDR-corrected at 0.05). Grand-average sorted and interpolated trials at electrode position Cz, time-locked to zeros in the car x position (*car zero*, black line). Time points of maximum car deviation from the road (*car max*) per trial are indicated with a red line; participant averages (grey) of the steering wheel signal and car x position with their corresp. Grand averages (black) are shown on top. ERD/ERS patterns, time-averaged across the epoch length as described, in four frequency bands of interest. All results are shown for **(A)** Execution and **(B)** Observation.

Similar analyses for the *Reactive Steering (Dist.)* condition are shown in Figures 7A,B; results for the remaining conditions are provided in the Supplementary Figures S2, S4, lower panels; Supplementary Figures S3, S5. Compared to the *Passive Steering* condition void of any error connotation, we observed clear frontocentral (Executors) and centroparietal (Observers) scalp distributions in the grand-average EEG signals. Overall, there appeared to be a frontal shift during task execution compared to simple task observation. Apart from the mentioned differences in comparison to the *Passive Steering* condition, additional significant differences arose in comparison with the *Proactive Steering* condition for both roles; however, no differences between the two *Reactive Steering* conditions were found. Considering the topographical maps, we chose to investigate the single-channel EEG activity at electrode position Cz in this case; the sorted and interpolated grand-average trials are shown in the image plots of Figures 7A,B. As can be seen, we observed strong modulations of Cz EEG activity with the car’s specific x position on the screen in the Executors’ data; a similar though much more attenuated behavior also translated to the Observers’ data.

As in the *Passive Steering* condition, we observed similar ERD/ERS patterns in the alpha band in the *Reactive Steering.* In addition, fronto-central delta band activity arose in the Executors, which could not be observed in the Observer data.

**Figure 8:**
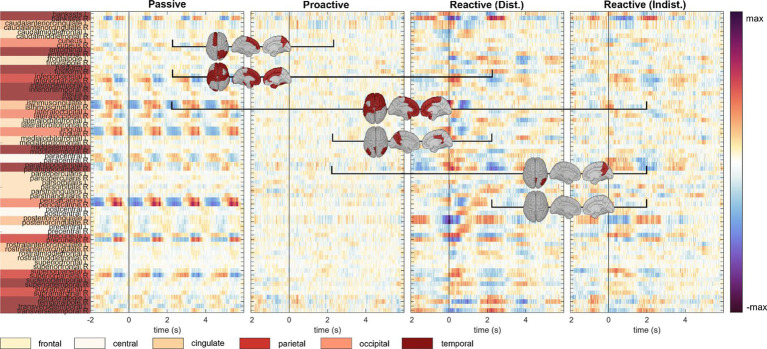
Observation, *car zero* time-lock. Grand-average cortical activity at 68 regions defined within the Desikan-Killiany atlas for *Passive, Proactive, and Reactive Steering (Dist./Indist.)* conditions (f.l.t.r.). Pairwise significant differences (Friedman-Nemenyi test, FDR-corrected at 0.01) are shown as dark red patches on the cortical maps.

**Figure 9:**
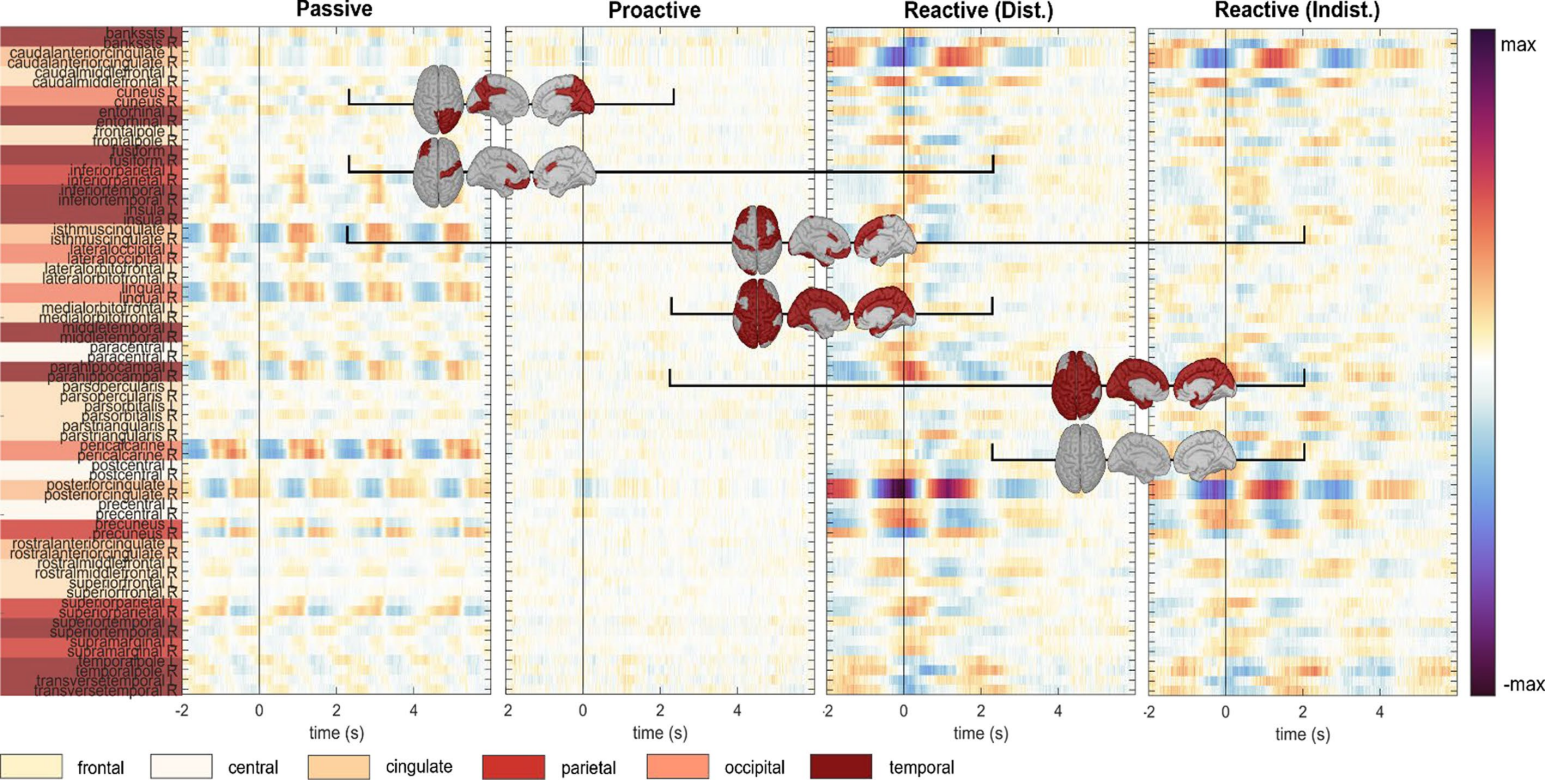
Execution, *car zero* time-lock. Grand-average cortical activity at 68 regions defined within the Desikan-Killiany atlas for *Passive, Proactive,* and *Reactive Steering (Dist./Indist.)* conditions (f.l.t.r.). Pairwise significant differences (Friedman-Nemenyi test, FDR-corrected at 0.01) are shown as dark red patches on the cortical maps.

In line with analyses regarding the *steer zero* time-lock (see previous section), we analyzed the cortical activity within each condition in the *car zero* time-lock for both Observer and Executor data; pairwise significant differences between conditions at 0.05 (FDR corrected) are once again shown as red areas on the cortical maps (Figures 8, 9, resp.). We observed very clear modulations with the car’s x position in the *Passive Steering* condition that largely coincided for both roles. In detail, grand-average signals in the Desikan-Killiany atlas revealed shared activity in the bilateral pericalcarine cortices, lingual gyri, the isthmus of the cingulate cortices, precunei, and the superior parietal gyri.

We interestingly observed stronger overall activity within the *Passive Steering* condition compared to other conditions in the Observers than in the Executors; however, we report increased activity in the posterior cingulate cortex for the Executors that was not found for Observers. As in the *steer zero* time-lock, little activity was found within the *Proactive Steering* condition; however, both roles showed increased engagement of the bilateral posterior and caudal anterior cingulate cortices, as well as the parahippocampal gyri, and temporal regions within both *Reactive Steering* conditions.

**Figure 10:**
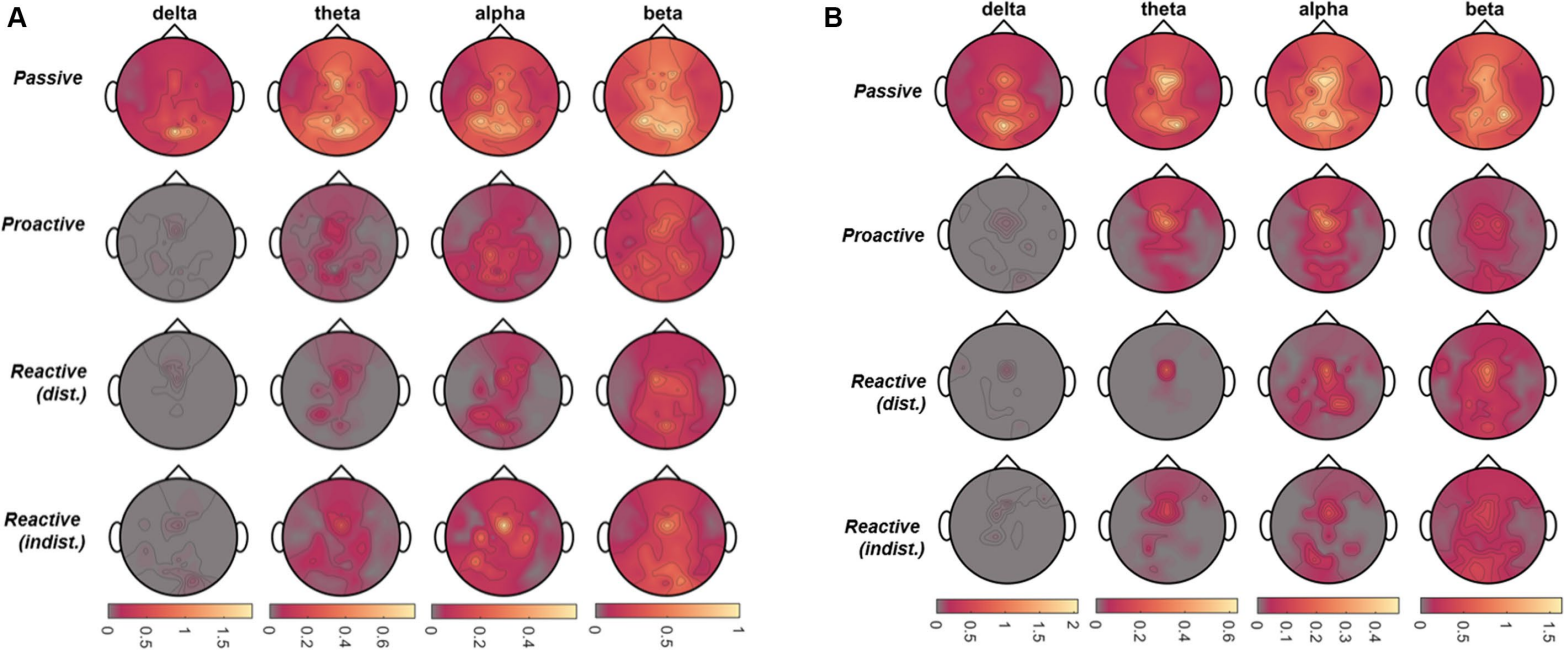
*Car zero* time-lock. Total information outflow per channel as obtained from the Directed Transfer Function (DTF) for *Passive, Proactive,* and *Reactive Steering (Dist./Indist.),* Steering conditions (f.t.t.b.) in four frequency bands of interest for **(A)** Observers and **(B)** Executors.

To estimate directional connectivity between distinct brain regions, we utilized the standardized participant averages of the form $X\in R^{M\times N\times S}$ in the *car zero* time-lock for both roles, with *M* = 55 channels, N=1601 time points [(−2,6)s epoch], and S=26 participants. The optimum model orders per condition and role were again exclusively found to be $p_{\mathrm{opti}}\in \left\{21,22\right\}$ . Topographical maps displaying the total information outflow per band (target-averaged DTF results) in the different paradigm conditions are shown in Figures 10A,B for Observers and Executors, respectively. As in the *steer zero* time-lock, we observed large-scale information outflow at central and parietal regions during the *Passive Steering* condition in all frequency bands, whereas the total information outflow within the other conditions increasingly condensed toward fronto-central sites with decreasing frequency.

### 3.3 Differences between observed and executed driving

**Figure 11:**
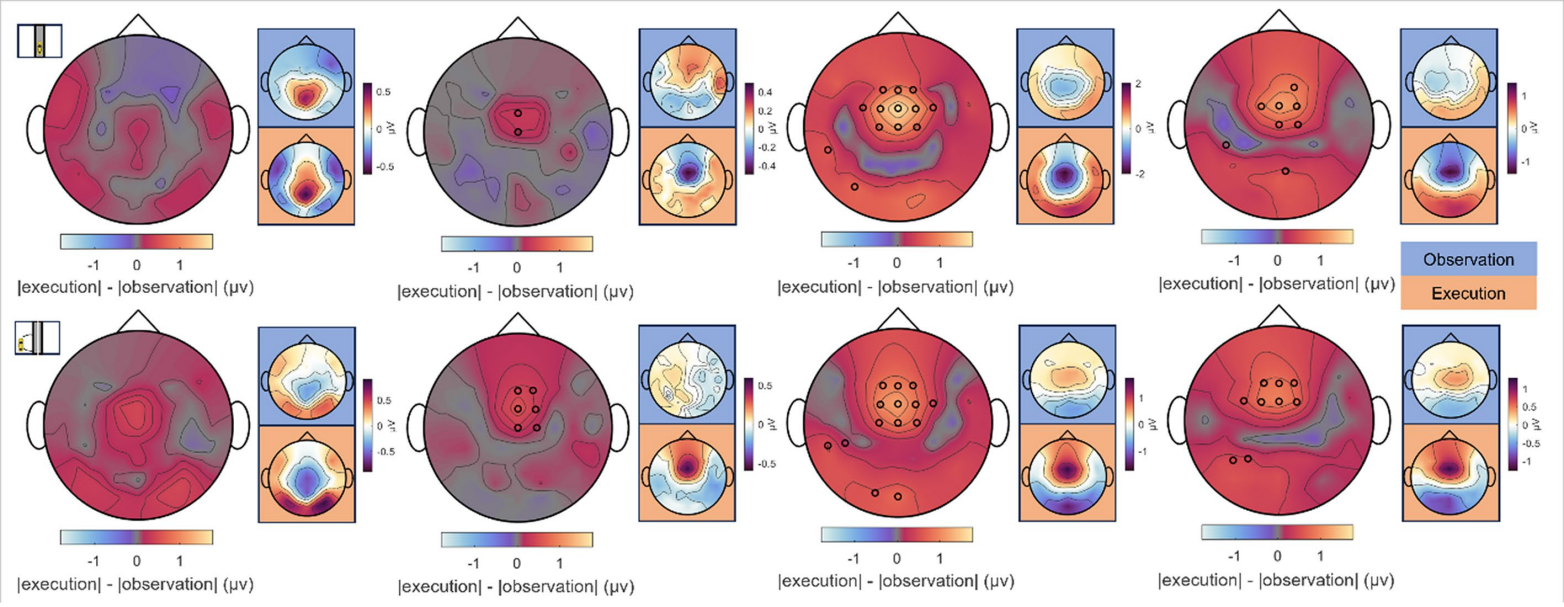
Differences between Observation and Execution, car time-locks. Absolute differences between Observation and Execution at the time points of (top row) zero and (bottom row) maximum car deviation from the screen’s center (*car zero* and *car max*) for *Passive, Proactive,* and *Reactive Steering (Dist./Indist.)* conditions (f.l.t.r.). Large topographical maps indicate the difference maps, smaller topographical maps highlighted in blue and orange correspond to the grand-average signals per condition for Observation and Execution, respectively. Channels displaying significant differences between both roles are indicated with small black circles (Wilcoxon signed rank test, FDR-corrected at significance level 0.05).

To analyze differences between observed and executed car driving within error-free and erroneous steering conditions at moments of zero and maximum car deviation from the center of the screen (*car zero* and *car max*), a Wilcoxon test along with FDR correction at a significance level of 0.05 was consulted. Considering these two time points of interest in four conditions and 55 EEG channels, we corrected for a total of N=440 tests. Interestingly, no significant differences arose for a comparison between Observer and Executor data in the *Passive Steering* condition for either time point of interest (see Figure 11, leftmost topographical maps). However, within the *Proactive* and *Reactive Steering* conditions, we observed significant differences between the roles at fronto-central electrode sites. Specifically in the *Reactive Steering (Dist.)* condition, an area of significance around the electrode position FCz emerged, which similarly arose for the *Reactive Steering (Indist.)* condition as well.

### 3.4 Regression results

**Figure 12:**
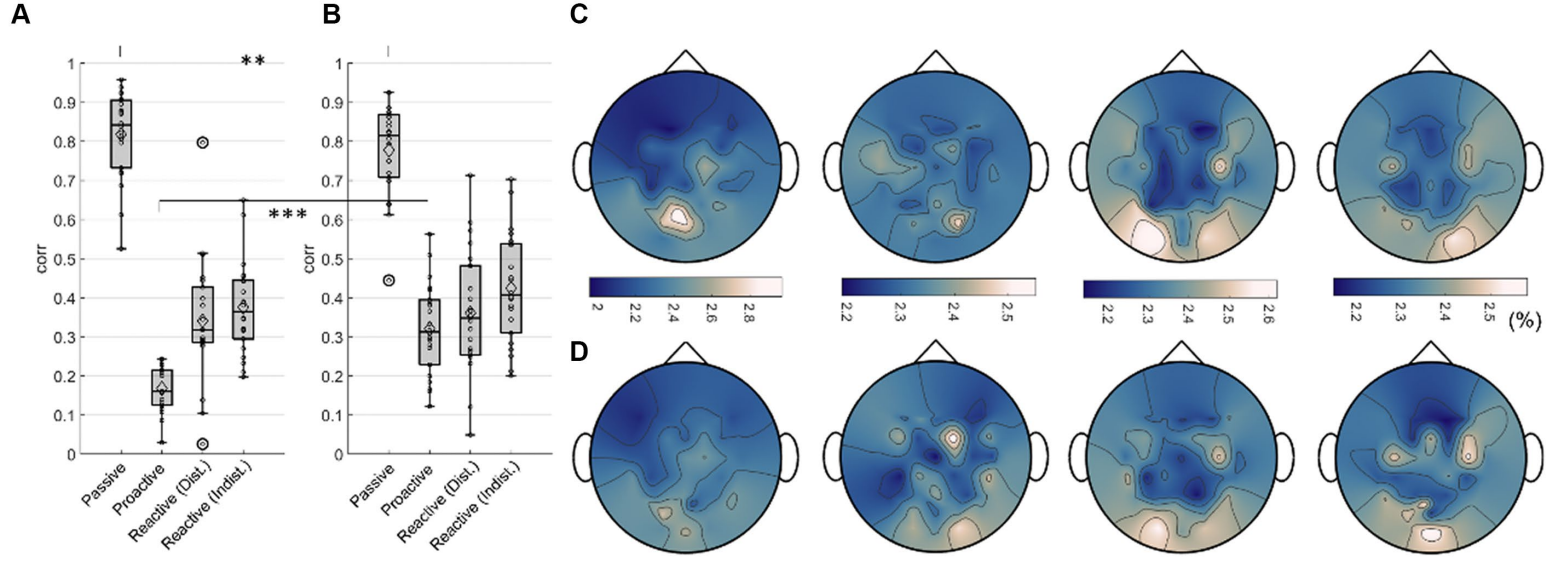
Regression results, Execution. Boxplots of maximum testing correlations obtained throughout all 100 epochs of training the model when regressing **(A)** the car’s x position and **(B)** the steering wheel signal. Small black dots indicate single participant maxima, diamonds denote the means, large circles indicate outliers. Significant differences (Wilcoxon signed rank test, FDR-corrected at 0.01) are marked with two asterisks (**). Averaged normalized saliency maps displaying each channel’s importance for regressing **(C)** the car’s x position and **(D)** the steering wheel signal.

We trained separate models for all participants in each condition to regress the car’s x position on the screen and the steering wheel signal from the Executors’ EEG data, respectively. The training curves of both the root mean square error and Pearson’s correlation coefficient for the regression of both signals are provided in the Supplementary Figures S10, S11. For both signals, the maximum testing correlation between signal and prediction is shown in Figure 12 (A: car position, B: steering wheel signal). A Wilcoxon signed rank test revealed significantly higher regression performance for the *Passive Steering* condition (FDR-corrected at 0.01), and significantly decreased performance for the *Proactive Steering* condition (FDR-corrected at 0.001) when regression the car’s x position compared to the steering wheel signal. No significant differences arose between both performances for the *Reactive Steering* conditions, although we observed generally higher maximum correlation values per participant when regressing the steering wheel signal (see Tables 1, 2). Notably, the performance considerably dropped for all conditions apart from the *Passive Steering* condition, where median values of 0.84 and 0.81 for Pearson’s correlation arose.

**Table 1:** Pearson correlation values (Corr r) for car position and steering wheel signal regression on the testing set.

	Observation						Execution					
	Car x position (px)			Steering signal (a.u.)			Car x position (px)			Steering signal (a.u.)		
	Mean (SD)	Median	Range	Mean (SD)	Median	Range	Mean (SD)	Median	Range	Mean (SD)	Median	Range
Passive	0.66 (0.17)**	0.68	[0.15, 0.93]	0.54 (0.20)**	0.61	[0.08, 0.84]	0.82 (0.12)**°°	0.84	[0.53, 0.96]	0.78 (0.12)**°°	0.81	[0.44, 0.93]
Proactive	0.14 (0.09)	0.14	[−0.03, 0.33]	0.12 (0.10)**	0.12	[−0.04, 0.27]	0.17 (0.16)**°°°**	0.16	[0.03, 0.24]	0.32 (0.12)**°°°	0.31	[0.12, 0.56]
Reactive (Dist.)	0.24 (0.11)	0.24	[0.09, 0.52]	0.20 (0.09)**	0.20	[0.06, 0.46]	0.35 (0.06)	0.32	[0.03, 0.80]	0.36 (0.16)**	0.35	[0.05, 0.71]
Reactive (Indist.)	0.23 (0.09)**	0.23	[0.05, 0.37]	0.22 (0.12)**	0.22	[0.03, 0.42]	0.38 (0.11)**	0.37	[0.20, 0.65]	0.42 (0.14)**	0.41	[0.20, 0.70]

** Significant differences between observation and execution, significance level 0.01.

°° / °°° Significant differences between car x position and steering (execution only), significance level 0.01 and 0.001.

**Table 2:** Root mean square error (RMSE) values for car position and steering wheel signal regression on the testing set.

	Observation						Execution					
	Car x position (px)			Steering signal (a.u.)			Car x position (px)			Steering signal (a.u.)		
	Mean (SD)	Median	Range	Mean (SD)	Median	Range	Mean (SD)	Median	Range	Mean (SD)	Median	Range
Passive	203 (40)	205	[113, 384]	0.26 (0.17)	0.21	[0.05, 0.70]	157 (42)	154	[81, 238]	0.19 (0.11)	0.15	[0.05, 0.41]
Proactive	12 (10)	8	[4, 43]	0.02 (0.01)	0.01	[0.01, 0.04]	10 (6)	8	[4, 27]	0.02 (0.01)	0.01	[0.01, 0.04]
Reactive (Dist.)	367 (75)	364	[243, 520]	0.49 (0.16)	0.45	[0.27, 0.80]	357 (81)	359	[214, 544]	0.47 (0.14)	0.44	[0.27, 0.76]
Reactive (Indist.)	326 (74)	302	[232, 457]	0.45 (0.20)	0.45	[0.13, 0.79]	316 (73)	305	[217, 462]	0.41 (0.18)	0.39	[0.13, 0.77]

** Significant differences between observation and execution, significance level 0.01.

°° / °°° Significant differences between car x position and steering (execution only), significance level 0.01 and 0.001.

An analysis of the importance of each channel unveiled largely identical grand average (normalized) saliency maps for both the regression of the car’s x position (Figure 12C) and the steering wheel signal (Figure 12D) in each condition (*Passive Steering, Proactive Steering, Reactive Steering (Dist.), Reactive Steering (Indist.),* f.l.t.r.). Overall, considerable feature importance lies on parietal and occipital channels for all conditions, with increased engagement of central channels during the *Proactive* and *Reactive Steering* conditions. Within the *Reactive Steering* conditions, we further observed increased channel importance at electrode positions C3 and C4.

## 4 Discussion

Within our analysis of both observation and execution data during a car driving simulation, we addressed three major questions. First, how does the brain manifest motor control across various steering conditions during executed car driving? Second, how do visual and feedback processing vary between error-free and error-prone driving performance in both observed and executed driving, and how do both roles differ from each other? And lastly, which non-biological signal – the car’s position on the screen or the steering wheel signal – is best represented in the EEG?

### 4.1 Cortical representations of passive, proactive, and reactive steering

To investigate brain signals relating to the steering process, we time-locked the EEG data to zero-crossings of the steering wheel signal. Within this time-lock, we observed largely coinciding scalp topographies across all steering conditions (*Passive, Proactive,* and *Reactive Steering*, Figure 3 and Supplementary file). Significant differences only arose in pairwise comparisons with the largely attenuated signals of the *Proactive Steering* condition, which most likely traces back to the minimal steering necessary during this condition. For all other conditions exhibiting comparable levels of steering movement, no significant differences arose, indicating matching executive activity in the brain that presumably relates to the steering motion itself and disregards error-free or error-prone driving performance. Nonetheless, we observed a qualitative frontal-shift within *Reactive Steering* compared to *Passive Steering* (large topographical maps, Figure 3B), substantiated by directional connectivity analyses unveiling increasingly focal fronto-central areas of the scalp as sites of highest information outflow during *Proactive* and *Reactive Steering* conditions. Source analysis traced these observations back to increased activity in the caudal anterior cingulate cortex (Figure 4, third and fourth panel), a region reportedly involved in the cognitive control of motor behavior (Holroyd et al., 2004) and performance monitoring (Ursu et al., 2009). In addition, increased activity in the bilateral parahippocampal gyrus, as well as the right caudal middle frontal gyrus and frontal pole arose; regions involed in visuospatial processing (Aminoff et al., 2013) and reorientation of attention (Japee et al., 2015) in humans, as well as evaluation of self-generated decisions in monkeys (Tsujimoto et al., 2010). However, shared regions of cortical activity could be found for all steering conditions (Figure 4). In this context, we specifically report engagement of the posterior cingulate cortex, which has been linked to both the integration of reward processing, attention, and motor control systems and the dynamic modification of strategy (Pearson et al., 2009). Furthermore, notable engagement of temporal regions arose, which we however hypothesize relates to the increased noise generated by the wheel during the conditions involving expansive steering motion (*Passive* and *Reactive Steering*).

Interestingly, we additionally observed differences in event-related desynchronization/synchronization (ERD/ERS) between the steering conditions. In detail, we observed prominent alpha and especially beta activity at electrode positions C3 and C4 during the perfectly periodic pre-defined car movement within the *Passive Steering* condition, both sites situated bilaterally above the hand regions of the sensorimotor cortex. In contrast, activity during the *Reactive Steering* conditions moved noticeably towards occipital regions especially in the alpha frequency band. This indicates increased emphasis on processing visual information within *Reactive Steering* conditions compared to the *Passive Steering* condition and aligns with previous findings of Garcia and colleagues, who likewise reported a transition from delta and beta activity during proactive brain states to prominent alpha activity during reactive brain states (Garcia et al., 2017).

Sorting all trials at electrode position FCz with respect to their steering latency, we further found clear modulations in the brain signals with the steering wheel signal for all conditions, with positivities in the EEG visibly coinciding with instances of maximum steering wheel deflection (image plots, Figure 3), coinciding with previous reports in the context of error processing by Burle et al. (2008) who showed similar fronto-central positivities in the EEG at instances of corrective button-presses.

### 4.2 Cortical representations of visual and feedback processing

Employing an additional time-lock to instances of zero crossings of the car’s x position on screen, we further analyzed brain responses phase-locked to the visual input delivered within our car-driving paradigms. As both Observers and Executors were exposed to the same visual stimulants, we investigated both the Observers’ and the Executors’ data within this time-lock.

Notably, the brain patterns matched closely between Observers and Executors within the *Passive Steering* condition (see Figure 6), substantiated by a lack of observed significant differences between the roles (Figure 11, leftmost topographical maps). Both roles showed strong modulations with the visual feedback (moving car) especially in parietal regions and displayed large-scale significant differences in fronto-central and occipital channels when compared to both *Reactive Steering* conditions (Figure 6), indicating a clear distinction between the processing of error-free and error-prone driving performance. Additionally, the ERD/ERS topographies (Figure 6, grey boxes) showed clear occipital engagement for both roles in the theta and alpha frequency bands, contrasting with the central (motor) activity observed in the steering time-lock (Figure 3, grey boxes).

As the error-free driving performance within the *Passive Steering* condition was entirely fake, the Executors’ steering movement within this condition did not correlate directly with the car’s position on-screen, yielding largely distinct patterns for both time-locks (compare Figures 3A, 6A) and thus both separate processes – motor activity and visual processing – in the brain. In contrast, the high-level correlations between steering and car position during the other conditions lead to matching scalp topographies in the Executors for both time-locks (compare Figures 3B, 7A), disallowing a separation of brain regions engaging for both individual processes. A comparison to the Observers’ data within *Proactive* and *Reactive Steering* revealed significant differences especially in the fronto-central areas of the scalp, presumably due to the slightly more attenuated and parietally shifted potentials during observation despite overall similar patterns (see Figure 11, second, third and fourth columns). The lack of a similarly significant difference during *Passive Steering* (first column) indicates that the observed disparities are not solely related to the additional motor activity within the Executors. This assumption is strengthened by findings of increased bilateral activity in the caudal anterior cingulate cortex (Holroyd et al., 2004; Ursu et al., 2009) within *Reactive Steering* conditions in the Executors in both the steering and the car position time-lock (see Figures 4, 8, 9), as well as more focal frontocentral sites of information outflow within out connectivity analyses (Figures 10A,B).

### 4.3 Representation of sub-processes in the brain

For both Observers and Executors, the *Passive Steering* condition yielded the highest correlations in our regression, most likely due to the perfectly periodic modulations evoked in the brain by the strict periodicity of the car movement within this condition. However, the synchrony between visual input and motor output within this steering condition may additionally contribute to high regression performances. In detail, the participants observed a periodically moving car programmed to always remain perfectly on the winding road. As such, target (the white centerline) and feedback (the moving car) concurred throughout the condition, allowing the focus of attention to condense strictly to the moving car. Interestingly, we nonetheless observed significantly higher performance for regressing the car position compared to the steering wheel signal in the *Passive Steering* condition within Executors, tentatively indicating a more prominent representation of the visual aspect of the task compared to the mostly automated steering (see Figure 12).

In contrast, the correlations within the *Reactive Steering* conditions merely reached half of the values for the *Passive Steering*, displaying no significance in the differences in the regression of both signals. This might suggest that both processes – visual feedback processing and motor control – commanded equal levels of related brain activity, yet the direct correlations between steering and car position within these conditions disallows a clear assessment. As the *Reactive Steering* conditions did not follow the strict periodicity of the *Passive Steering* condition, the observed drop in performance was to be expected to a certain degree. However, within *Reactive Steering*, the car continuously deviated from the road, constantly splitting the participants’ attention between the intended and the observed target (car) position, which might have additionally led to lower correlations. Furthermore, while a regression of the car position corresponded simultaneously to a regression of the intended position on-screen during the *Passive Steering* condition, it rather equaled a regression of the continuous feedback error (the deviation from the road) in the case of the *Reactive Steering* condition. As such, the results are indeed interesting despite the moderate correlations and indicate the feasibility of regressing continuous error signals as observed within our previous work (Pulferer et al., 2023) even during real-life situations.

Lastly, we observed the lowest correlations when regressing within the *Proactive Steering* condition, presumably due to both the minimal motor output and the barely visible deviations from the road. Nonetheless, we obtained significantly higher correlations when regressing the steering wheel signal, implying that the minute deviations from the road provided less relevant information than even the minimal steering movement.

### 4.4 Limitations

Despite the exploratory nature of the current work to analyze the recently reported continuous correlates to error processing in more detail, several limitations of the present study need to be mentioned.

First and foremost, as has been the modus operandi for most studies reporting on electroencephalographic correlates in the past decades (Larson and Carbine, 2017), prior sample size calculations were neglected. As a result, the exact statistical power of the observed effects cannot be stated; a shortcoming which needs to be alleviated in follow-up studies.

Secondly, the lengthy measurement sessions and restricting nature of the paradigm during revoked steering agency gave rise to various superimposing factors; specifically, fatigue, frustration, and boredom may have impacted the recorded cortical information – a recurring issue in neuroscientific studies which nonetheless remains problematic. In addition, the limited spatial resolution within source projection methods further demands conservative interpretation.

Thirdly, the utilized car driving paradigm was displayed in a most minimalistic fashion, only incorporating a bird view of the lane instead of immersive driving simulations. While this reduction of distractors proved to be necessary for interpreting our first results presented in the current work, future work will need to close the gap to more realistic driving experiences to observe similar effects in a real-world scenario.

And lastly, the approach used for investigating the feasibility of regressing both the steering and the car position signals via a neural network could serve only as a proof-of-concept. Different architectures would need to be consulted in future work to obtain a reliable estimate for the optimum performance obtainable within this research question.

## 5 Conclusion

We conclude that a clear separation of distinct subprocesses constituting car driving utilizing different time-locks is feasible, yet additional data of observed driving remains crucial in obtaining a clear distinction between motor activity and visual / higher-level cognitive processing. We reported both increased fronto-central brain activity and increasingly focal fronto-central information outflow during execution, as well as significant fronto-central differences between observed and executed driving, for error-prone driving performance, which traces back to increased performance monitoring within the caudal anterior cingulate cortex. Depending on the time-locking signal of either the steering signal or the car position, we further observed clear event related desynchronization and synchronization above the hand motor area (beta band) and visual cortex (alpha band), respectively. Lastly, we presented first results for regressing continuous road deviations from the EEG, indicating possible use-cases for investigating run-off-road scenarios. Overall, the results indicate that different subprocesses of car driving can be identified via brain recordings during both task execution and observation, and that cortical information related to continuous error processing can indeed be detected non-invasively for possible use in more naturalistic and intuitive control mechanisms in future brain-computer interface designs.

## Data availability statement

The raw data supporting the conclusions of this article will be made available by the authors, without undue reservation.

## Ethics statement

The studies involving humans were approved by the Ethics Board of Graz University of Technology. The studies were conducted in accordance with the local legislation and institutional requirements. The participants provided their written informed consent to participate in this study.

## Author contributions

HP: Writing – review & editing, Writing – original draft, Visualization, Methodology, Investigation, Formal analysis, Data curation, Conceptualization. CG: Writing – review & editing, Supervision, Resources, Project administration, Methodology. GM-P: Writing – review & editing, Visualization, Supervision, Project administration, Methodology, Funding acquisition, Conceptualization.
