# Supplementary material for: Investigating multilevel cognitive processing within error-free and error-prone feedback conditions in executed and observed car driving
Source: Front Hum Neurosci. 2024 Jun 27;18:1383956. doi: 10.3389/fnhum.2024.1383956 (PMC11236611; doi:10.3389/fnhum.2024.1383956)
Supplement: Supplementary file 1 [file Data_Sheet_1.PDF]

Supplementary Material

1 Supplementary Figures

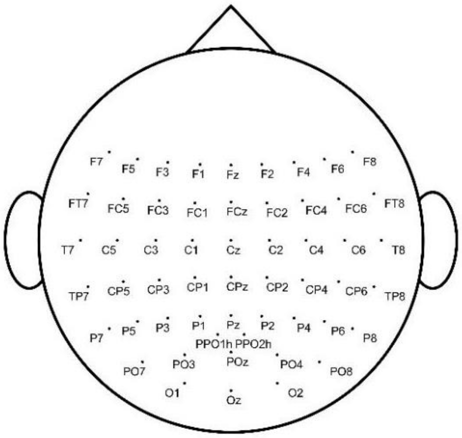

Supplementary Figure S1. Channel locations. Modified 10-10 system setup with increased signal density in the parietooccipital region.

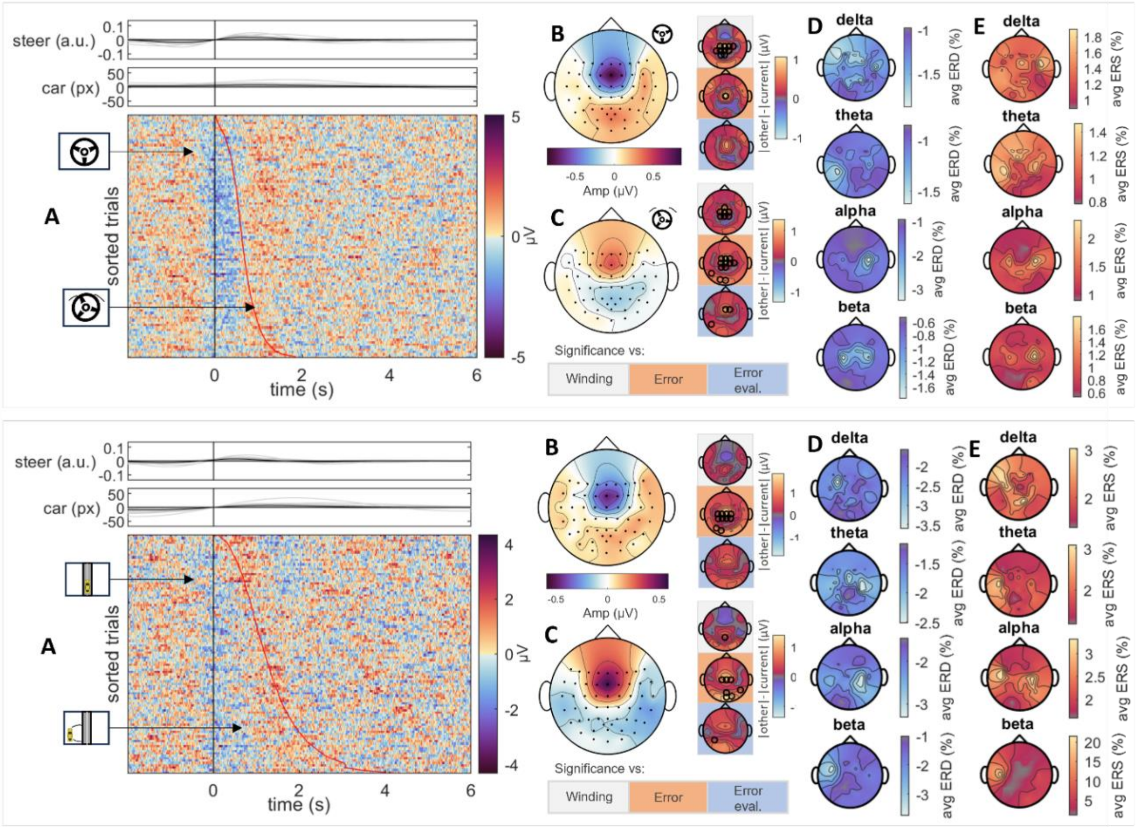

Supplementary Figure S2. Proactive Steering. Execution. (A) Grand-average sorted and interpolated trials at electrode position FCz, time-locked (black line) to zeros in the car x position (car zero, bottom panel) and steering signal (steer zero, top panel). Time points of maximum car deviation from the road (car max) / maximum steering wheel deflection (steer max) per trial are indicated with a red line; participant averages (grey) of the steering wheel signal and car x position with their corresp. grand averages (black) are shown on top. Grand average topographical maps at (B) the time-lock (car zero / steer zero) and (C) maximum car deviation / steering wheel deflection. Pairwise significant differences to the other conditions are shown as black circles

in the adjacent topographical maps (Friedman-Nemenyi test, FDR-corrected at 0.05). (D) ERD and (E) ERS patterns, time-averaged across the epoch length as described, in four frequency bands of interest.

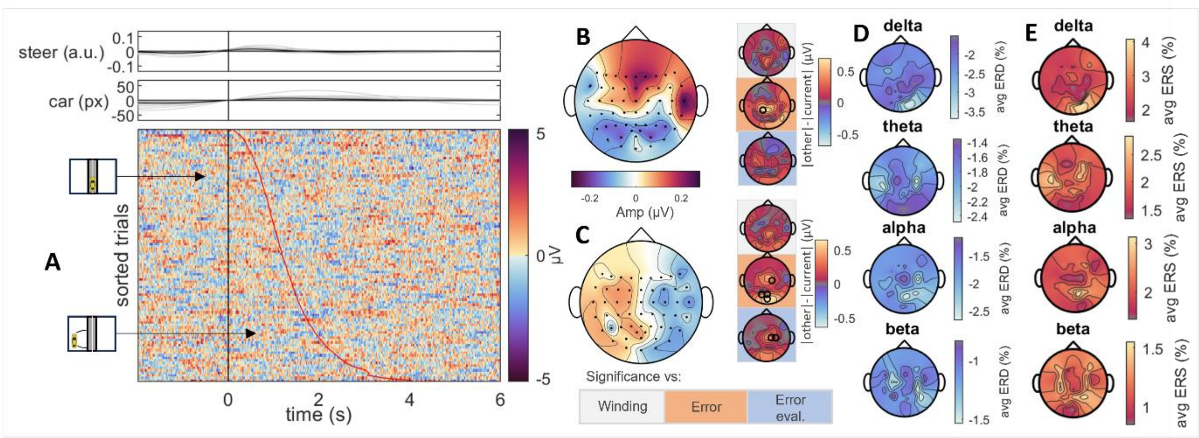

Supplementary Figure S3. Proactive Steering. Observation. (A) Grand-average sorted and interpolated trials at electrode position FCz, time-locked (black line) to zeros in the car x position (car zero). Time points of maximum car deviation from the road (car max) per trial are indicated with a red line; participant averages (grey) of the steering wheel signal and car x position with their corresp. grand averages (black) are shown on top. Grand average topographical maps at (B) the time-lock (car zero) and (C) maximum car deviation. Pairwise significant differences to the other conditions are shown as black circles in the adjacent topographical maps (Friedman-Nemenyi test, FDR-corrected at 0.05). (D) ERD and (E) ERS patterns, time-averaged across the epoch length as described, in four frequency bands of interest.

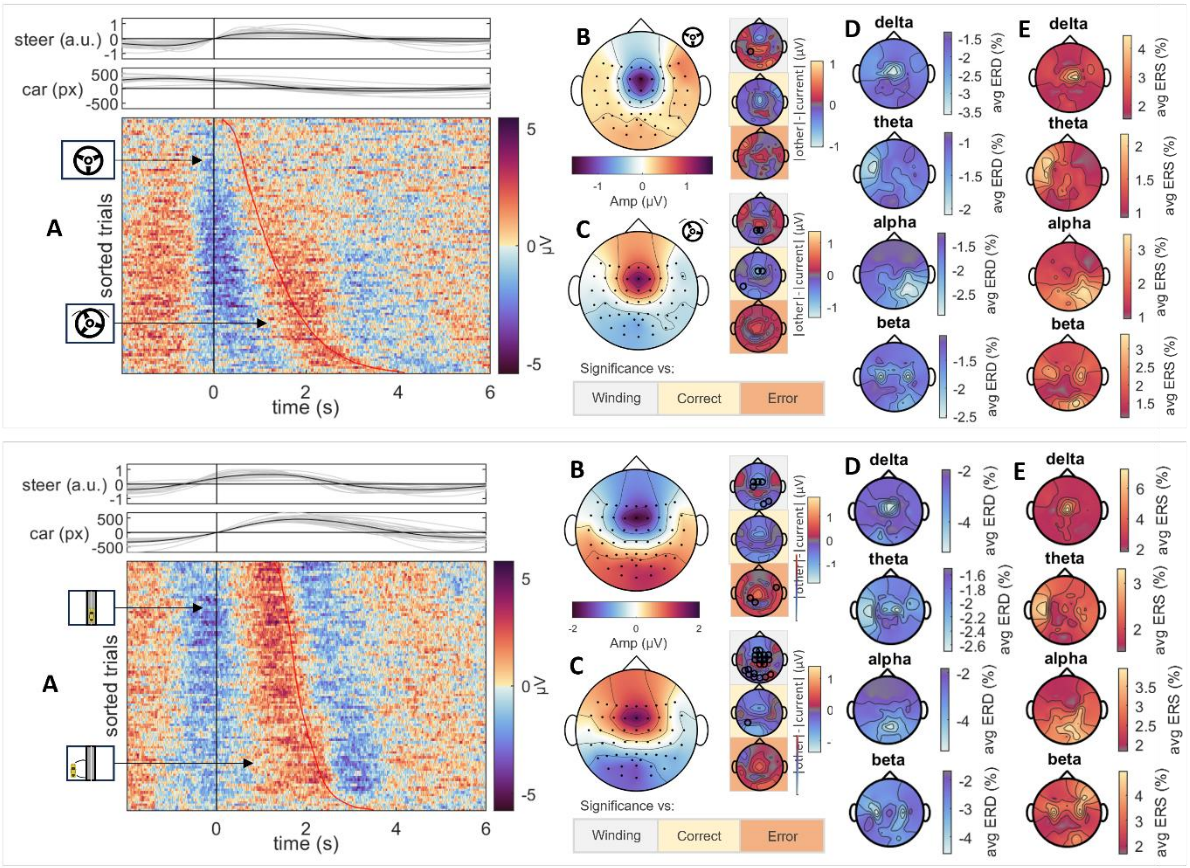

Supplementary Figure S4. Reactive Steering (Indist.). Execution. (A) Grand-average sorted and interpolated trials at electrode position FCz, time-locked (black line) to zeros in the car x position (car zero, bottom panel) and steering signal (steer zero, top panel). Time points of maximum car deviation from the road (car max) / maximum steering wheel deflection (steer max) per trial are indicated with a red line; participant averages (grey) of the steering wheel signal and car x position with their corresp. grand averages (black) are shown on top. Grand average topographical maps at (B) the time-lock (car zero / steer zero) and (C) maximum car deviation / steering wheel deflection. Pairwise significant differences to the other conditions are shown as black circles in the adjacent topographical maps (Friedman-Nemenyi test, FDR-corrected at 0.05). (D) ERD and (E) ERS patterns, time-averaged across the epoch

length as described, in four frequency bands of interest.**Supplementary Figure S2.** Channel locations. Modified 10-10 system setup with increased signal density in the parietooccipital region.

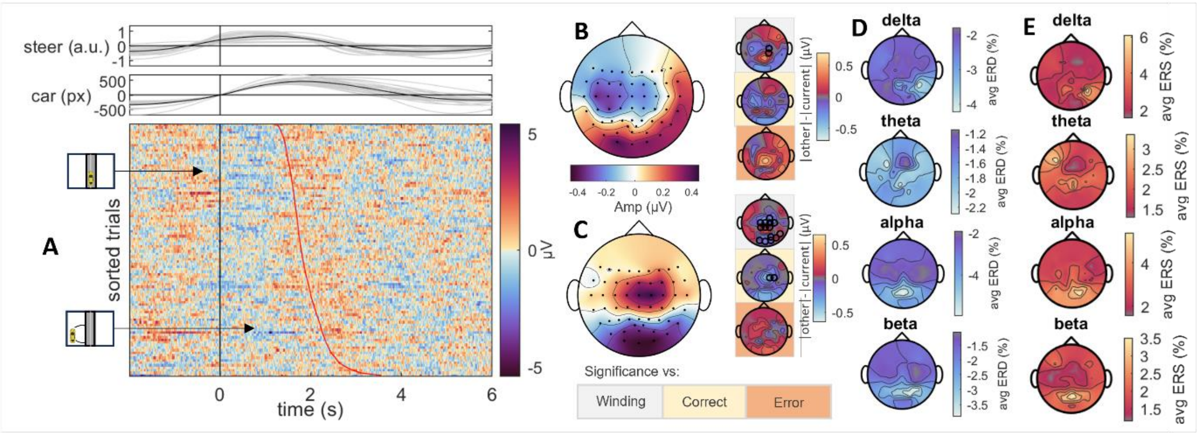

Supplementary Figure S5. Reactive Steering (Indist.). Observation. (A) Grand-average sorted and interpolated trials at electrode position Cz, time-locked (black line) to zeros in the car x position (car zero). Time points of maximum car deviation from the road (car max) per trial are indicated with a red line; participant averages (grey) of the steering wheel signal and car x position with their corresp. grand averages (black) are shown on top. Grand average topographical maps at (B) the time-lock (car zero) and (C) maximum car deviation. Pairwise significant differences to the other conditions are shown as black circles in the adjacent topographical maps (Friedman-Nemenyi test, FDR-corrected at 0.05). (D) ERD and (E) ERS patterns, time-averaged across the epoch length as described, in four frequency bands of interest.**Supplementary Figure S3.** Channel locations. Modified 10-10 system setup with increased signal density in the parietooccipital region.

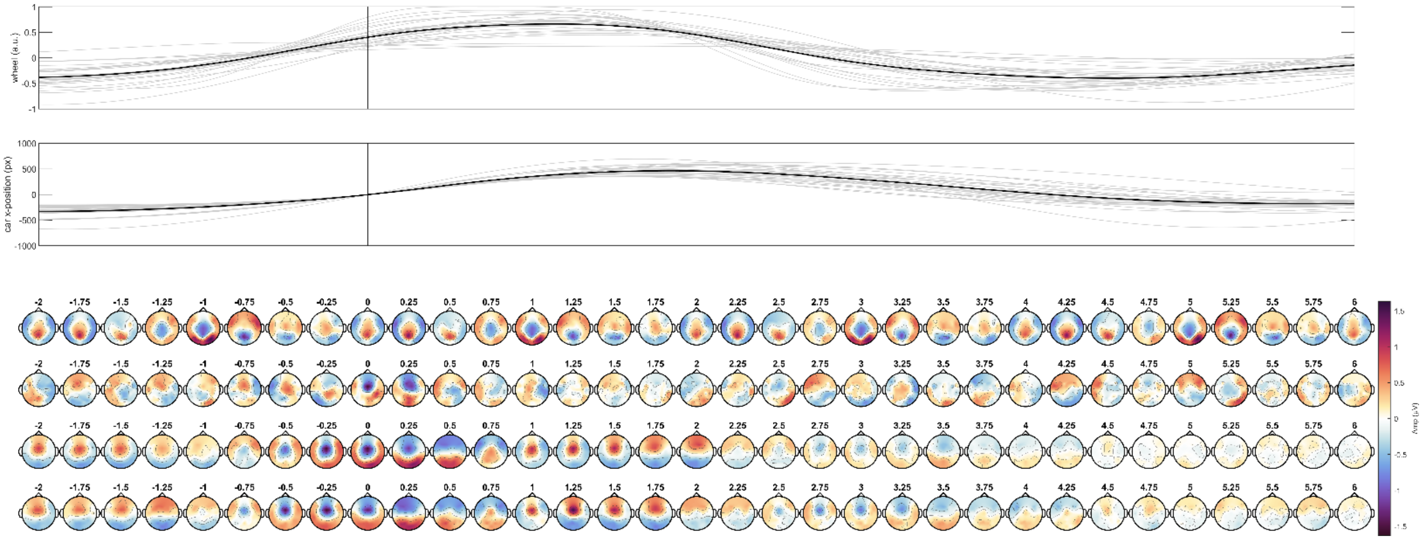

Supplementary Figure S6. Car zero time-lock. Execution. Grand average steering wheel signal and car x-position (black) and subject averages (grey) between [-2,6]s of the time-lock (top panels). Topographical maps for Passive, Proactive, and Reactive Steering (Dist./Indist.) conditions (f.t.t.b.).

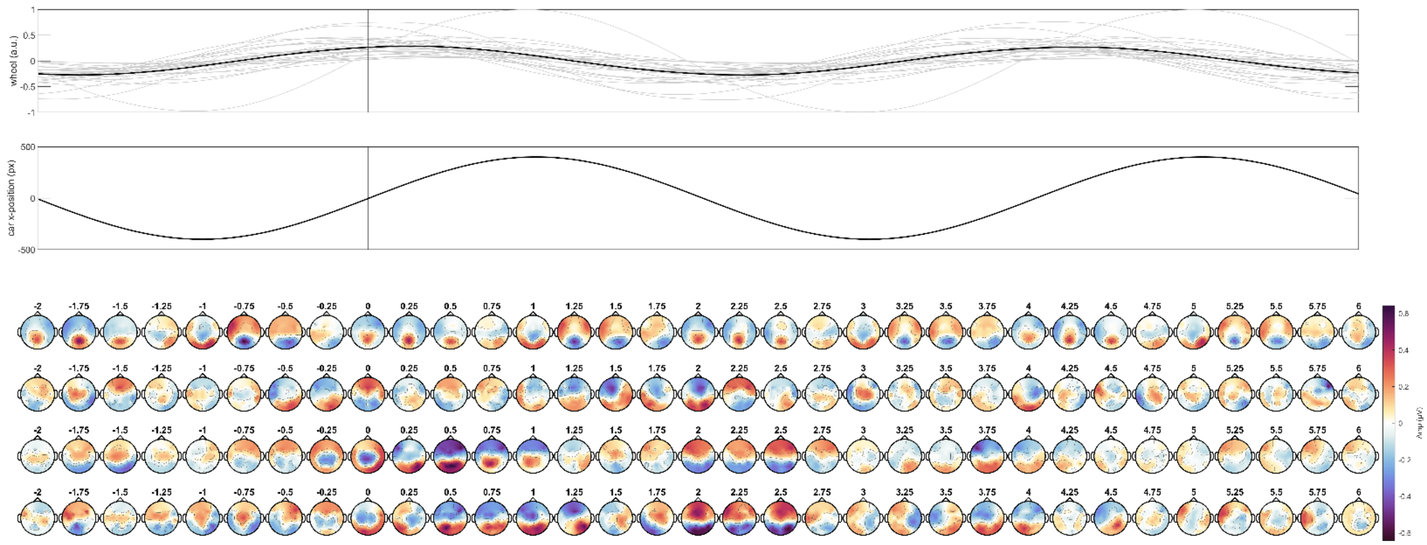

Supplementary Figure S7. Car zero time-lock. Observation. Grand average steering wheel signal and car x-position (black) and subject averages (grey) between [-2,6]s of the time-lock (top panels). Topographical maps for Passive, Proactive, and Reactive Steering (Dist./Indist.) conditions (f.t.t.b.).

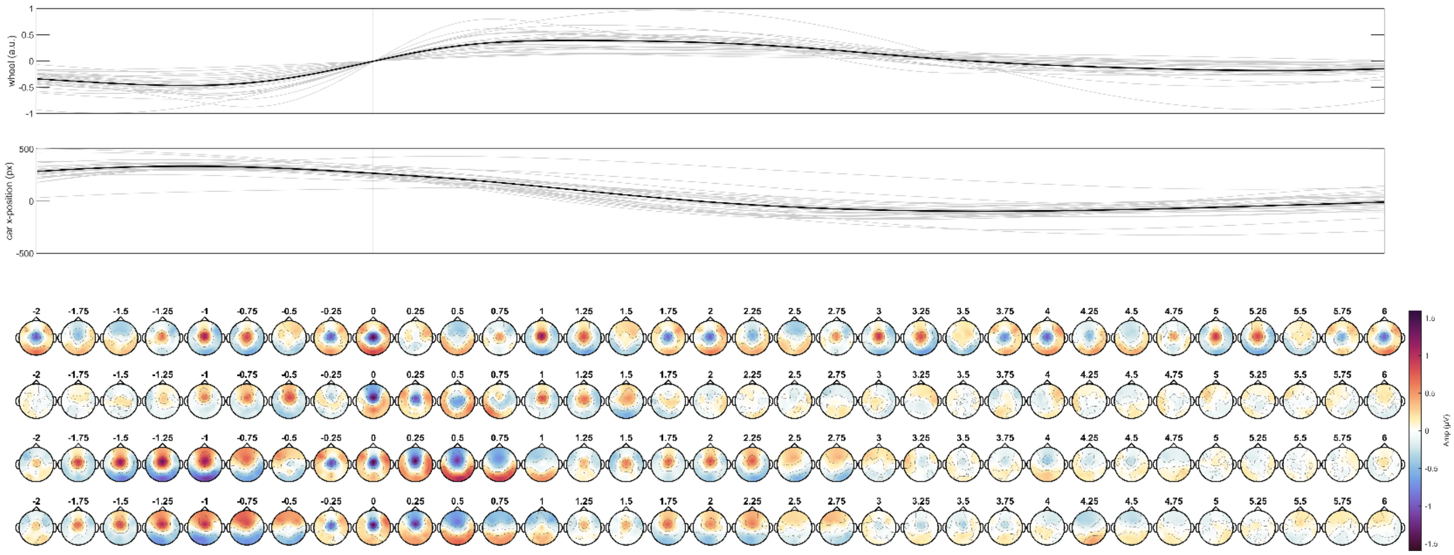

Supplementary Figure S8. Steer zero time-lock. Execution. Grand average steering wheel signal and car x-position (black) and subject averages (grey) between [-2,6]s of the time-lock (top panels). Topographical maps for Passive, Proactive, and Reactive Steering (Dist./Indist.) conditions (f.t.t.b.).

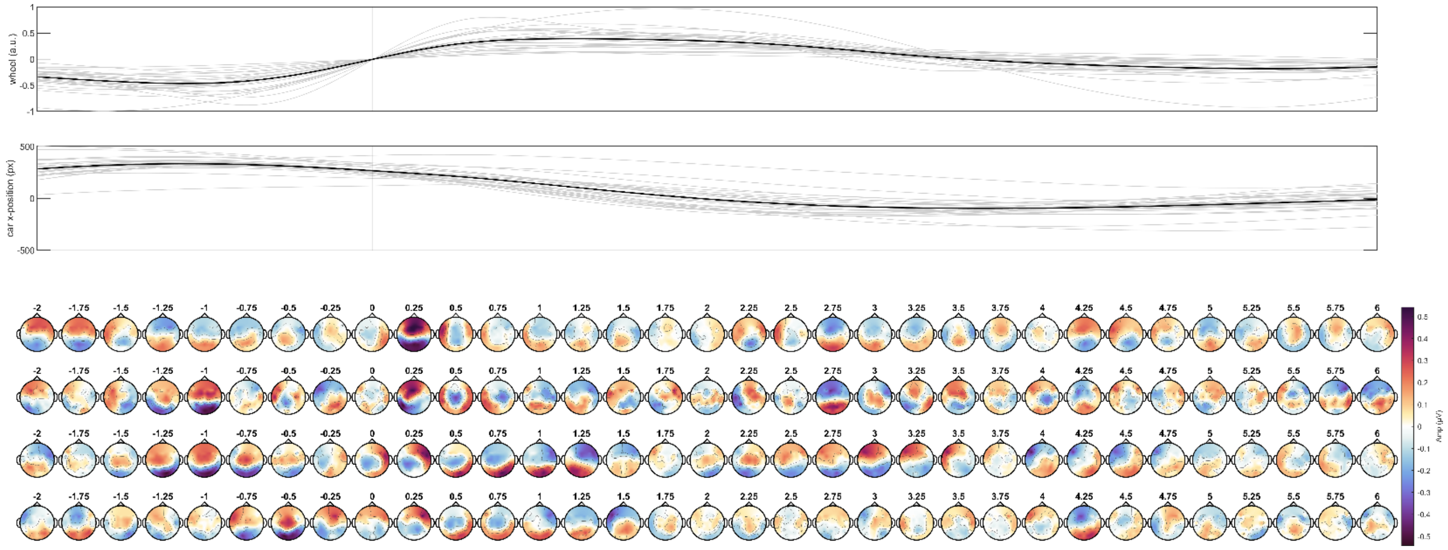

Supplementary Figure S9. Steer zero time-lock. Observation. Grand average steering wheel signal and car x-position (black) and subject averages (grey) between [-2,6]s of the time-lock (top panels). Topographical maps for Passive, Proactive, and Reactive Steering (Dist./Indist.) conditions (f.t.t.b.).

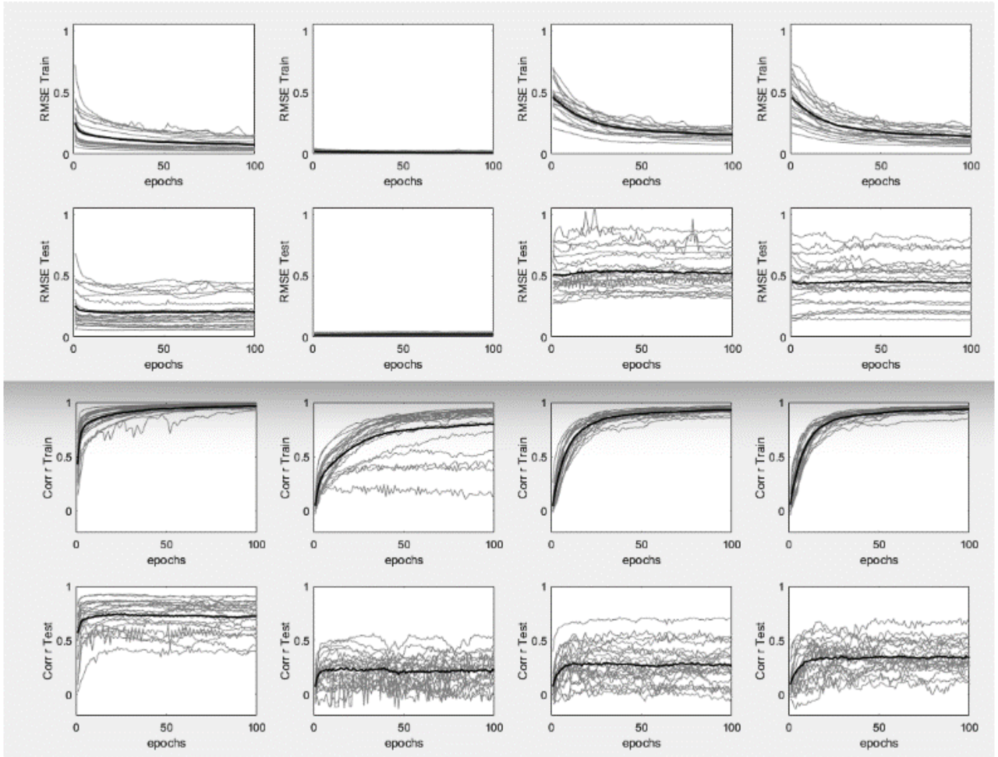

Supplementary Figure S10. Performance metrics over training epochs, steering signal regression. Root-mean-square error (a.u., first and second row), and Pearson correlation (third and fourth row) for training and test set, respectively. Columns (f.l.t.r.) correspond to Passive, Proactive and Reactive Steering (Dist./Indist.).

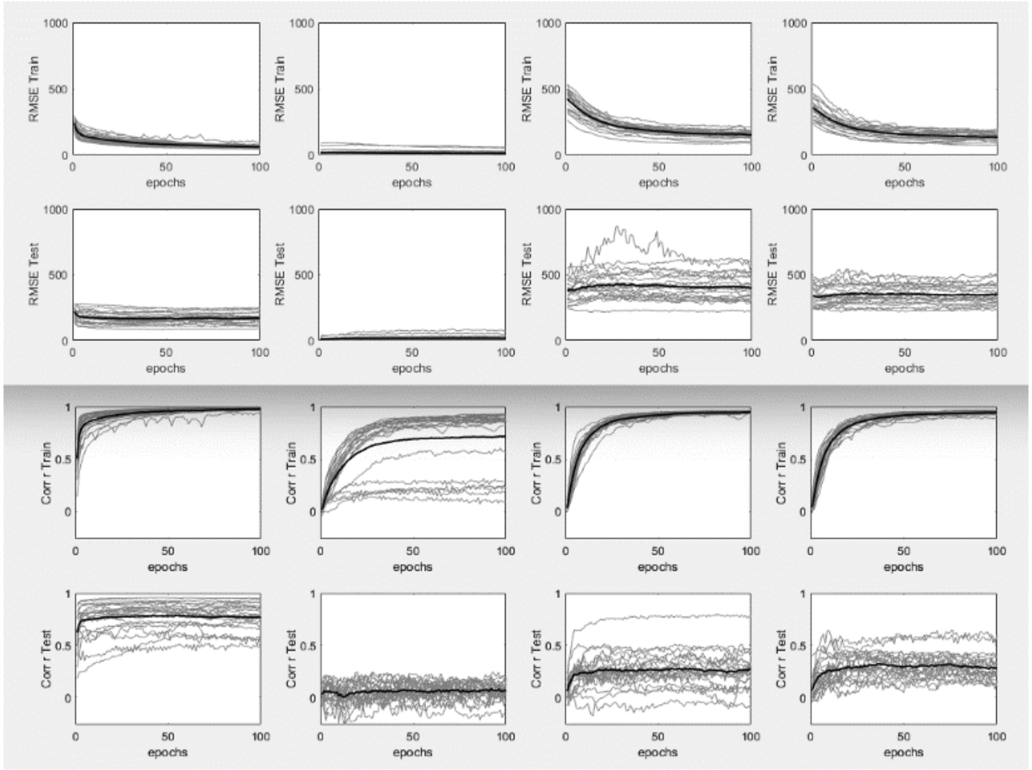

Supplementary Figure S11. Performance metrics over training epochs, car x-position regression. Root-mean-square error (a.u., first and second row), and Pearson correlation (third and fourth row) for training and test set, respectively. Columns (f.l.t.r.) correspond to Passive, Proactive and Reactive Steering (Dist./Indist.).

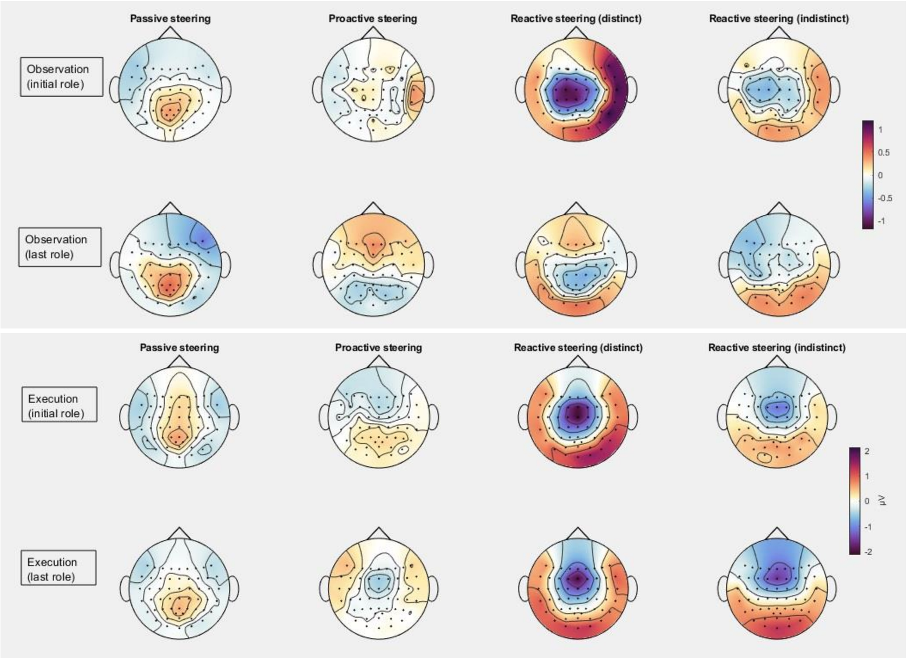

Supplementary Figure S12. Comparison between initial and last performance of a role (i.e., participants assigned the Executor/Observer role first (initial role) versus participants assigned the Executor/Observer role last (last role)) for all four conditions, **car-zero timelock**.

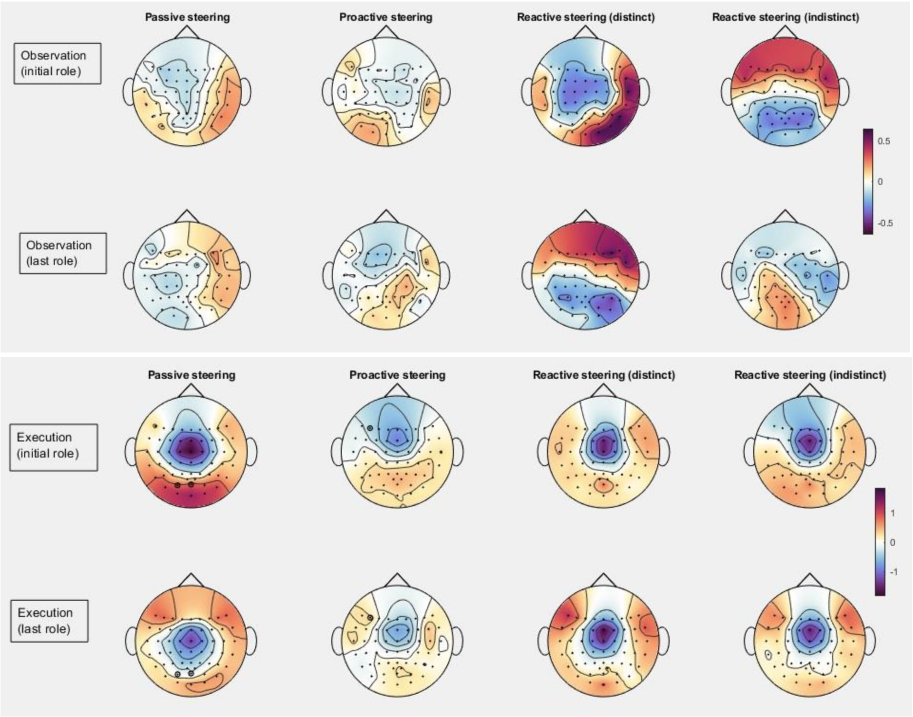

Supplementary Figure S13. Comparison between initial and last performance of a role (i.e., participants assigned the Executor/Observer role first (initial role) versus participants assigned the Executor/Observer role last (last role)) for all four conditions, **steer-zero timelock**. Significant differences are shown as black rings around the relevant electrodes.
